# Dual molecular therapy targeting tumor cell heterogeneity improves therapeutic efficacy in glioblastoma

**DOI:** 10.1016/j.isci.2025.113456

**Published:** 2025-08-28

**Authors:** Shuichiro Hirano, Atsuhito Uneda, Yoshihiro Otani, Yasuki Suruga, Ryoji Imoto, Madoka Hokama, Tsuyoshi Umeda, Ryosuke Ikemachi, Shohei Nishigaki, Nobushige Tsuboi, Keigo Makino, Naoya Kemmotsu, Yasuhito Kegoya, Yuji Matsumoto, Yusuke Tomita, Yosuke Shimazu, Joji Ishida, Kentaro Fujii, Hiroaki Wakimoto, Shota Tanaka, Isao Date

**Affiliations:** 1 Department of Neurological Surgery, Okayama University Graduate School of Medicine, Dentistry, and Pharmaceutical Sciences, 2-5-1 Shikata-cho, Kita-ku, Okayama 700-8558, Japan; 2 Department of Neurosurgery, Mitsui Memorial Hospital, 1 Kanda-Izumi-cho, Chiyoda-ku, Tokyo 101-8643, Japan; 3 Department of Neurosurgery and Neuroendovascular Surgery, Hiroshima City Hiroshima Citizens Hospital, 7-33 Moto-machi, Naka-ku, Hiroshima 730-8518, Japan; 4 Department of Neurosurgery, Okayama Kyokuto Hospital, 567-1 Kurata, Naka-ku, Okayama 703-8265, Japan; 5 Department of Neurosurgery, Massachusetts General Hospital, Harvard Medical School, Boston, MA 02114, USA

**Keywords:** Pharmacology, Natural sciences, Biological sciences, Cancer

## Abstract

The intratumoral heterogeneity of glioblastoma, comprising glioblastoma stem cells (GSCs) and differentiated glioblastoma cells (DGCs), contributes to treatment resistance. We explored combination therapy targeting both GSCs and DGCs. Candidate drugs predicted to be highly effective against GSCs and DGCs were identified through ***in silico* screening**, which utilized antitumor efficacy data of therapeutic agents and gene expression profiles of cancer cell lines. IC50 values of the candidate drugs were determined using *in vitro* cell proliferation assays. Belinostat and Dasatinib were found to be the most effective against GSCs and DGCs, respectively. Their combination showed synergistic effects *in vitro*. Transcriptome analysis revealed the suppression of the G2/M transition and the PI3K-Akt-mTOR signaling pathway following combination therapy. Histological analysis confirmed reduced proliferation and increased apoptosis. *In silico* screening successfully identified candidate drugs for GSCs and DGCs. These results suggest that the dual targeting of GSCs and DGCs may help overcome intratumoral heterogeneity in glioblastoma.

## Highlights

• Glioblastoma heterogeneity, involving GSCs and DGCs, contributes to resistance to treatment
• *In silico* screening identified Belinostat and Dasatinib as effective against GSCs and DGCs
• The combination showed synergistic effects and suppressed key pathways *in vitro* and *in vivo*
• Dual targeting may overcome glioblastoma heterogeneity and improve therapeutic outcomes

## Graphical abstract

**Figure:**
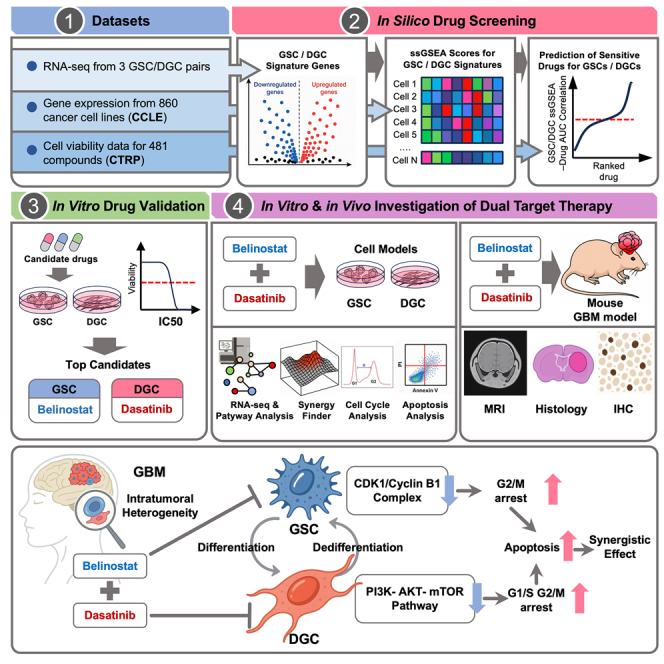

## Introduction

Glioblastoma (GBM) is the most aggressive malignant tumor originating in the brain parenchyma and has a poor prognosis. Despite current standard treatment composed of maximal safe resection followed by chemotherapy and radiotherapy, the median overall survival is less than 2 years.^1^^,^^2^ Nearly all patients have recurrence of tumors. Therefore, the development of new treatment is urgently needed, and several novel therapies, including immunotherapy,^3^ virotherapy,^4^ and molecularly targeted therapy,^5^ have been investigated.

The intratumoral heterogeneity of tumor cells is a hallmark of GBM and is considered to play a role in GBM resistance to conventional treatment.^6^ Cancer stem cell (CSC) theory serves as a valuable model for understanding the cellular and molecular underpinnings of GBM, and the existence of glioblastoma stem cells (GSCs) has been validated.^7^ The role of GSCs was extensively investigated, and GSCs are an attractive therapeutic target.^8^^,^^9^ Recent studies have reported the interactions of tumor cells with tumor cells or other cells such as immune cells, vascular cells, glial cells, and neurons. Wang et al. reported the interaction between GSCs and differentiated glioblastoma cells (DGCs), which participated in tumor growth and proliferation.^10^ We also reported the interaction between DGCs and macrophages via the Hippo pathway and CCN1, which contributed to the immune-suppressive tumor microenvironment in GBM.^11^ Clinical trials have investigated several pharmacological treatments, including monotherapy and combination therapy for GBM; however, no drug other than temozolomide has shown a survival benefit so far.^1^^,^^12^ In addition, although several agents targeting GSC-associated surface biomarkers or GSC-specific pathways were investigated, none of them showed a survival benefit in randomized clinical trials.^13^^,^^14^^,^^15^ Moreover, GSCs reversibly alter their status into DGCs.^16^ Thus, these findings suggested the importance of dual therapy targeting both GSCs and DGCs.

In this study, we proposed rational combinations of therapeutic agents targeting both GSCs and DGCs. This is based on the hypothesis that more effective antitumor effects can be obtained by selecting and combining drugs that are considered effective for each of the major constituent cells of the tumor, GSCs and DGCs. To identify candidate drugs for GSCs and DGCs, we calculated GSC and DGC gene expression signatures and adapted them to the publicly available gene expression data from systemic cancer cells, including brain tumors, and the drug sensitivity data of each cancer cell. This *in silico* screening successfully identified drugs that were effective against GSCs or DGCs. Moreover, the combination therapy of those drugs significantly improved therapeutic efficacy *in vitro* and *in vivo*.

## Results

### Selecting candidate drugs for combination therapy based on cancer cell line gene expression data and cell viability information for therapeutic compounds

**Figure 1:**
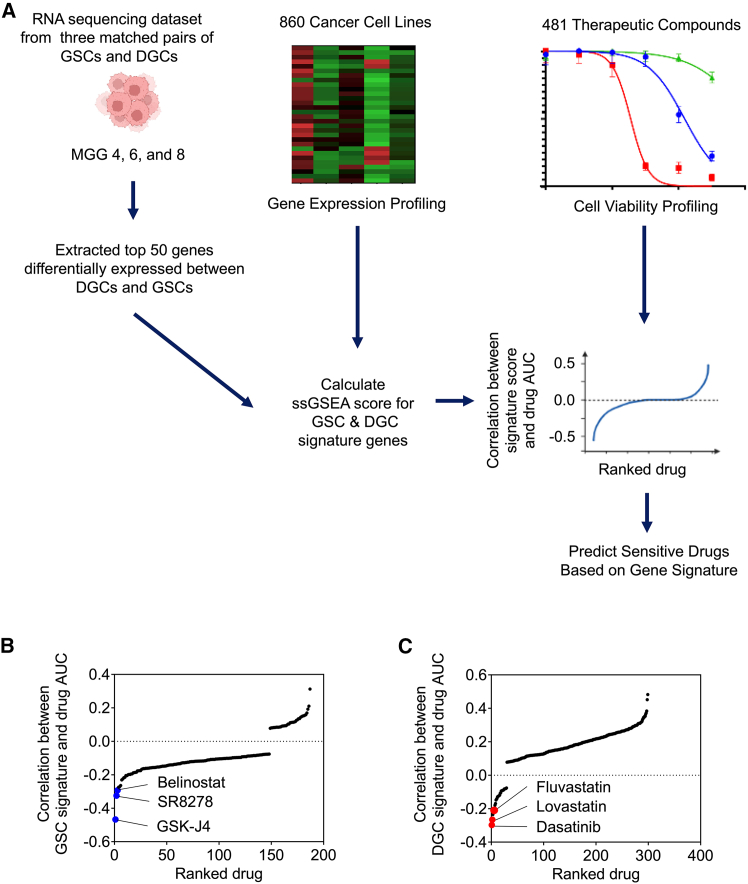
*In silico* screening revealed candidate drugs for targeting GSCs or DGCs (A) Schematic flow of the process to determine drugs that are predicted to be highly effective to GSCs and DGCs based on CCLE and CTRP data. (B) The dot plot shows a significant correlation between the GSC signature score and treatment response. Blue dots indicate the top three drugs predicted to be highly potent. (C) The dot plot shows a significant correlation between the DGC signature score and treatment response. Red dots indicate the top three drugs predicted to be highly potent.

In our previous research, we analyzed a deposited RNA sequencing dataset from three matched pairs of GSCs and DGCs (MGG4, 6, and 8) (GEO: GSE54791).^11^^,^^16^ The extracted top 50 genes that differentially expressed between DGCs and GSCs were defined as DGC and GSC signature genes (Tables S1 and S2). We calculated ssGSEA scores of the DGC and GSC signature genes for cancer cell lines based on the gene expression data obtained from the CCLE (Table S3). To determine candidate drugs that would be effective for GSCs or DGCs, we calculated correlation coefficients between the GSC or DGC signature scores and AUC curves obtained from CTRP that tested 481 therapeutic compounds (Figure 1A; Tables S4, S5, and S6). Six drugs that showed the lowest correlation were determined as candidate drugs, which were GSK-J4, SR8278, and belinostat for the GSC signature score, and dasatinib, lovastatin, and fluvastatin for the DGC signature score (Figures 1B and 1C).

### Belinostat and dasatinib had the lowest IC50s as drugs targeting glioblastoma stem cells and differentiated glioblastoma cells, respectively

**Figure 2:**
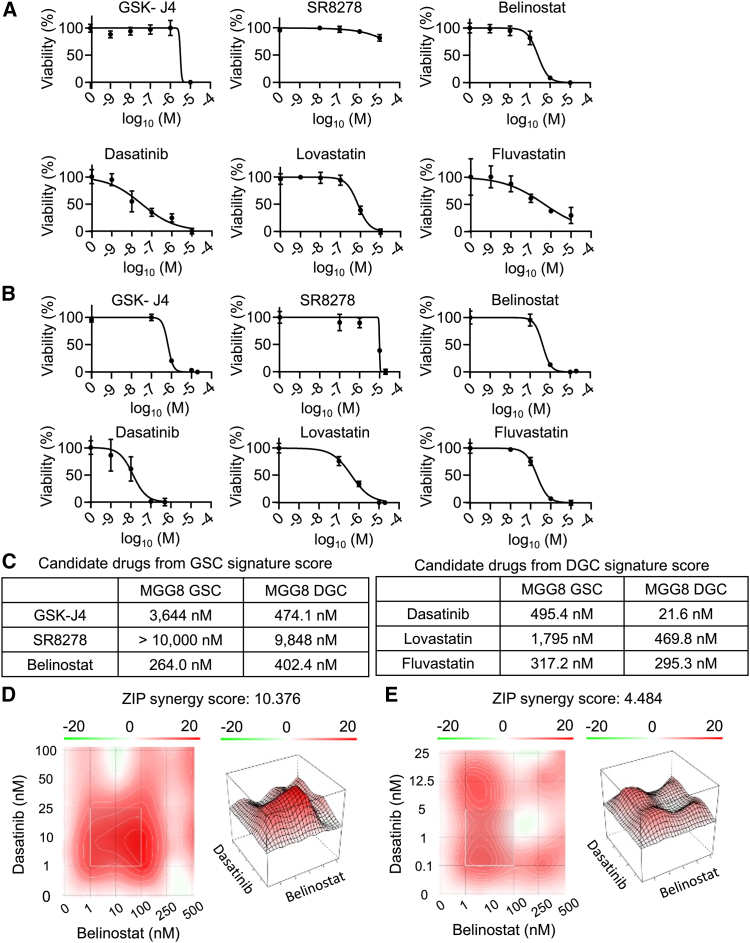
*In vitro* cell proliferation assay identified belinostat or dasatinib as GSC- or DGC-targeting drugs (A) Cell proliferation assay with GSC-targeting candidates in MGG8 GSCs (*n* = 5/each drug). (B) Cell proliferation assay with DGC-targeting candidates in MGG8 DGCs (*n* = 5/each drug). (C) Summary of IC50 for each drug. (D) Synergy score and synergy map for belinostat and dasatinib for MGG8 GSCs. (E) Synergy score and synergy map for belinostat and dasatinib for MGG8 DGCs.

To confirm the potency and selectivity of each drug for either GSCs or DGCs, IC50s were determined in two different patient-derived glioblastoma cell lines (MGG8 and MGG4). While some drugs, such as dasatinib, showed consistently higher sensitivity in DGCs as expected, other compounds exhibited cell line–dependent differences in drug response. For instance, GSK-J4, presumed to be effective against GSCs, showed limited efficacy in MGG8 GSCs. Lovastatin demonstrated greater sensitivity in DGCs than in GSCs in MGG8, but this trend was not observed in MGG4. These results suggest that drug responses vary depending on the specific cell line (Figures 2A–2C and S1A–S1C). Based on the IC50 data, tyrosine kinase inhibitor dasatinib and histone deacetylase (HDAC) inhibitor belinostat were considered to be the potent therapeutics targeting DGCs and GSCs, respectively (Figures 2C and S1C), and the effect of the combination therapy of dasatinib and belinostat was evaluated. The synergistic score was 4.484 for DGCs, which was additive, and 10.376 for GSCs, which was synergistic (Figures 2D and 2E). To assess the toxicity of each drug on normal cells, the IC50 for NHAs was determined. Notably, the IC50s for both belinostat and dasatinib against NHAs were higher than those against GSCs and DGCs (Figures S2A and S2B).

### Dual target therapy effectively suppressed the G2/M checkpoint and PI3K-Akt-mTOR signaling pathway

**Figure 3:**
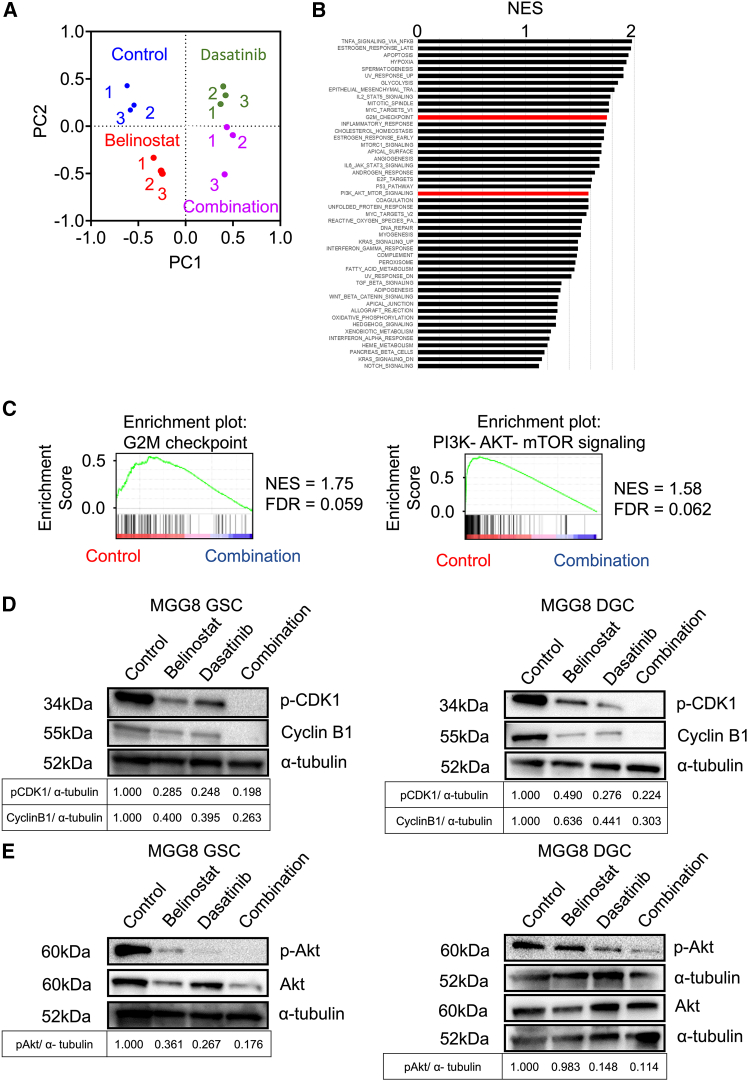
Combination therapy induced G2/M arrest and PI3K-Akt-mTOR pathway inhibition (A) Principal component analysis of transcriptome data in each therapy showed distinct groups (*n* = 3/each group). (B) Hallmark gene sets downregulated in combination therapy compared with the control. (C) Gene set enrichment analysis showed enrichment of the G2/M transition and PI3K-Akt-mTOR pathway in the control compared with combination therapy. (D) Western blot of Cyclin B1 and *p*-CDK1 regulating the G2/M transition in MGG8 GSCs and DGCs. Quantitative evaluation was performed, and the relative expression values are shown below the figures. (E) Western blot of Akt and *p*-Akt in MGG8 GSCs and DGCs. Quantitative evaluation was performed for *p*-Akt, and the relative expression values are shown below the figures.

To reveal the functional mechanisms of combination therapy, unbiased transcriptome analysis was performed using MGG8 GSCs treated with control, belinostat, dasatinib, or combination therapy. Principal component analysis showed that each treatment group had a distinct pattern (Figure 3A).

For further detailed analysis, altered pathways were investigated. In the belinostat group, the genes regulating the cell cycle were suppressed, especially the G2/M transition (Figures S3A and S3B). In contrast, genes related to the G2/M transition as well as the PI3K-AKT-mTOR pathway downstream of Src targeted genes were suppressed by dasatinib (Figures S3C and S3D). Furthermore, the G2/M transition and PI3K-Akt-mTOR pathway, which were suppressed in the monotherapy groups, were further suppressed in the combination group. GSEA showed the enrichment of the G2/M transition and PI3K-Akt-mTOR pathway in the control group compared with the combination group (Figures 3B and 3C). These results indicated that the G2/M transition and PI3K-AKT-mTOR pathway were the main targets in combination therapy.

### Dual target therapy suppressed phosphorylated-CDK1, cyclin B1, and phosphorylated-Akt

To confirm the alterations in the G2/M transition and the PI3K-Akt-mTOR pathway, western blotting was performed (Figures 3D and 3E). Quantitative evaluation results showed that the expression of phosphorylated CDK1, Cyclin B1, and phosphorylated Akt was significantly reduced in the combination group in both GSCs and DGCs of MGG8 (Figures S4A and S4B).

In MGG4, we also evaluated expression changes using western blot analysis. Among the factors regulating G2/M transition in GSC, the expression of phosphorylated CDK1 increased in all treatment groups. The expression of cyclin B1, which forms a complex with CDK1, was not suppressed by Dasatinib but was suppressed by combination therapy. Phosphorylated Akt showed no significant changes when compared between the control group and each treatment group. In DGCs, the expression of Cyclin B1, phosphorylated CDK1, and phosphorylated Akt was downregulated by drug administration, and these changes were enhanced by drug combination therapy (Figures S4C and S4D).

### Dual target therapy induced cell-cycle arrest at the G2 phase

**Figure 4:**
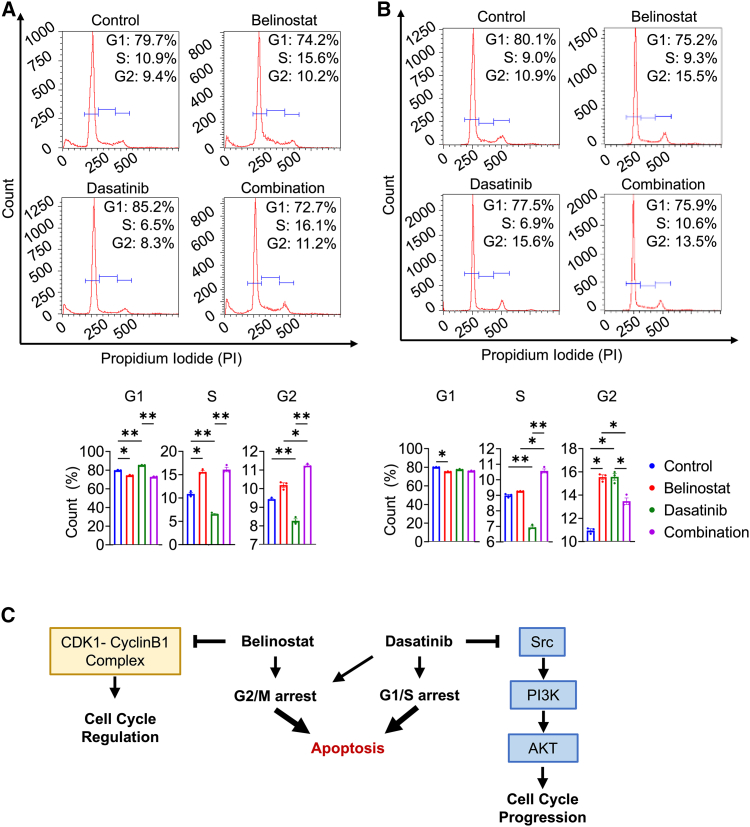
Combination therapy induced cell arrest at the G2 phase in GSCs and the S phase in DGCs (A) Cell cycle analysis of MGG8 GSCs treated with vehicle (control), belinostat, dasatinib, and combination (*n* = 3/each group). (B) Cell cycle analysis of MGG8 DGC treated with vehicle (control), belinostat, dasatinib, and combination (*n* = 3/each group). (C) Schema shows the effects of belinostat and dasatinib on the cell cycle. The results are shown as the mean ± standard error of the mean (SEM) of three independent experiments. ∗*p* < 0.05, ∗∗*p* < 0.01, ns: not significant, one-way analysis of variance (ANOVA) with Tukey’s multiple comparisons test.

To confirm the role of combination therapy in the cell cycle, MGG8 GSCs and DGCs were treated with either belinostat or dasatinib alone or in combination, and then the cell cycle was evaluated. In GSCs, there was a significant decrease in the G1 phase and an increase in the S phase in the belinostat group. In contrast, a slight decrease in the S and G2 phase was noted in the dasatinib group. In the combination group, a significant increase in the G2 phase was observed, which suggested G2/M arrest (Figure 4A). In DGCs, belinostat significantly decreased the G1 phase and increased the G2 phase, and dasatinib significantly decreased the S phase and increased the G2 phase. In the combination group, a significant increase in the S phase was observed compared with the other groups (Figure 4B). The effects of belinostat and dasatinib on the cell cycle are shown in Figure 4C.

### Combination therapy increased apoptotic cells *in vitro*

**Figure 5:**
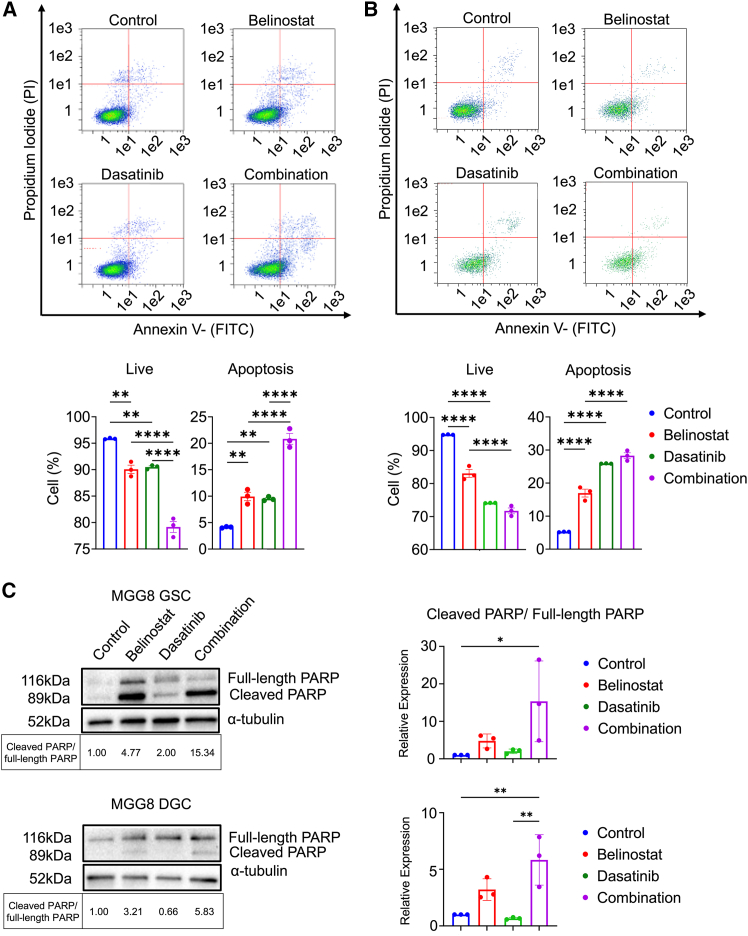
Combination therapy potently induced apoptosis in GSCs and DGCs (A) Apoptosis assay of MGG8 GSCs treated with vehicle (control), belinostat, dasatinib, and combination (*n* = 3/each group). (B) Apoptosis assay of MGG8 DGCs treated with vehicle (control), belinostat, dasatinib, and combination (*n* = 3/each group). (C) Western blot of full-length PARP and cleaved PARP in MGG8 GSCs and DGCs. Quantitative evaluation was performed, and relative expression values are shown as bar graphs with average values at the bottom of the figure. Cleaved PARP/full-length PARP ratios were calculated for each treatment group and normalized to the control group value. The results are shown as the mean ± standard error of the mean (SEM) of three independent experiments. ∗∗*p* < 0.01, ∗∗∗*p* < 0.001, ∗∗∗∗*p* < 0.0001, ns: not significant, one-way analysis of variance (ANOVA) with Tukey’s multiple comparisons test.

Since cell-cycle arrest causes apoptosis, Annexin V/PI staining was conducted to evaluate the cytotoxic effects. Apoptosis was evaluated as the sum of early and late apoptosis. The fraction of apoptotic cells in MGG8 GSCs, which was 4.1% in the control group, was significantly increased to 9.9% in the belinostat group and 9.5% in the dasatinib group. In the combination group, the fraction was 20.8%, which was a significant increase compared to the monotherapy groups (Figure 5A). In DGCs, the fraction of apoptotic cells significantly increased from 5.2% in the control group to 17.0% in the belinostat group, 25.9% in the dasatinib group, and 28.3% in the combination group (Figure 5B). We also performed an apoptosis assay by Annexin V/PI staining in MGG4 cells, and confirmed that the drug combination increased the proportion of apoptotic cells in both GSCs and DGCs (Figures S5A and S5B). To confirm the apoptosis in treatment groups, the expression of cleaved PARP was evaluated. In both MGG8 and MGG4, the expression of cleaved PARP was upregulated in the combination group in both GSCs and DGCs (Figures 5C and S5C).

### Dual target therapy reduced cell proliferation and increased apoptotic cells *in vivo*

**Figure 6:**
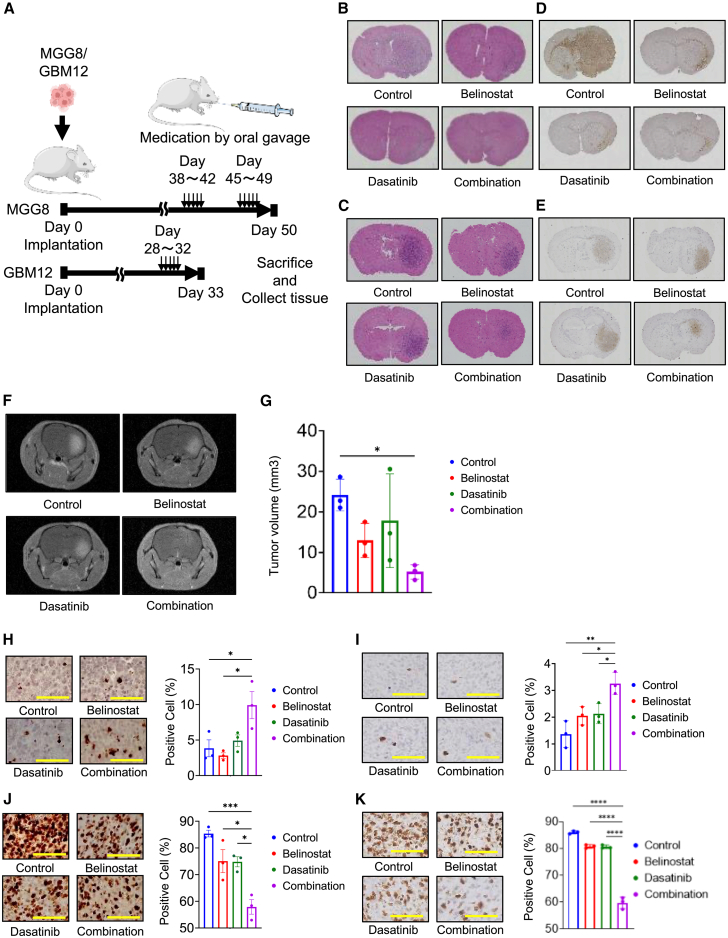
Combination therapy increased apoptosis and decreased cell proliferation in glioblastoma *in vivo* (A) Schema showing the *in vivo* assay. (B) Representative images of hematoxylin and eosin staining using tissues from each treatment group of MGG8. (C) Representative images of hematoxylin and eosin staining using tissues from each treatment group of GBM12. (D) Representative images of immunostaining with anti-HLA Class I antibody using tissues from each treatment group of MGG8. (E) Representative images of immunostaining with anti-HLA Class I antibody using tissues from each treatment group of GBM12. (F) Representative images of gadolinium contrast–enhanced MRI of GBM12-bearing mice at day 33 from each group. (G) Tumor volume of GBM12-bearing mice measured by MRI (*n* = 3/each group). (H) Immunostaining with anti-cleaved caspase-3 using tissues from each treatment group of MGG8 (3 mice per treatment group; 3 measurements per mouse). (I) Immunostaining with anti-cleaved caspase-3 using tissues from each treatment group of GBM12 (3 mice per treatment group; 3 measurements per mouse). (J) Immunostaining with anti-Ki-67 using tissues from each treatment group of MGG8 (3 mice per treatment group; 3 measurements per mouse). (K) Immunostaining with anti-Ki-67 using tissues from each treatment group of GBM12 (3 mice per treatment group; 3 measurements per mouse). The results are shown as the mean ± standard error of the mean (SEM) of three independent tissues. ∗*p* < 0.05, ∗∗∗*p* < 0.001, ∗∗∗∗*p* < 0.0001, one-way analysis of variance (ANOVA) with Tukey’s multiple comparisons test. The yellow bar in each figure indicates 100 μm.

To confirm the therapeutic activity *in vivo*, two tumor-bearing intracranial models, MGG8 and GBM12, were utilized, as shown in Figure 6A. In both models, hematoxylin and eosin staining (H.E) and immunohistochemistry for HLA (as a human-specific antigen) showed a reduction of tumor burden and invasion in the treatment groups compared to the control group (Figures 6B–6E). MRI was performed for the GBM12 model. A significant reduction in tumor size was observed in the combination group compared to the control group (24.1 mm^3^ and 5.2 mm^3^ in the control group and combination group, respectively) (Figures 6F and 6G). The rate for cleaved caspase-3–positive cells in MGG8 tumors was 3.8%, 2.8%, and 4.9% in the control, belinostat, and dasatinib groups, respectively, and there was no difference among them. The combination group showed a significant increase in cleaved caspase-3–positive cells (9.9%, Figure 6H). In GBM12 tumors, the rate for cleaved caspase-3–positive was 1.4%, 2.1%, and 2.1% in the control, belinostat, and dasatinib groups, respectively. The combination group showed a significant increase in cleaved caspase-3–positive cells (3.3%, Figure 6I). Conversely, the percentage of Ki-67-positive cells in MGG8 tumors was 85.4%, 75.1%, and 74.8% in the control, belinostat, and dasatinib groups, respectively. The combination group showed a significant decrease in the Ki-67-positive cell rate (57.9%) compared to the other groups (Figure 6J). In the GBM12 model, the percentage of Ki-67-positive cells was 86.1%, 80.8%, and 80.6% in the control, belinostat, and dasatinib groups, respectively. The combination group showed a significant decrease in the Ki-67-positive cell rate (59.7%) compared to the other groups (Figure 6K).

## Discussion

CSC theory was first reported in leukemia and is currently widely adapted to various tumor types, including GBM. The characteristic features of CSCs contribute to the failure of cancer treatment, recurrence, heterogeneity, resistance to traditional therapies, and avoidance of immunological surveillance. Therefore, CSCs are considered the most critical target for treatment.^17^ In GBM, several agents targeting GSC-associated surface biomarkers or GSC-specific pathways have been investigated. For example, Notch signaling plays an important role in the maintenance of GSCs and therapeutic resistance, and γ-secretase inhibitors showed antitumor activity in preclinical and early trials.^18^^,^^19^ In contrast, no drug targeting GSCs has shown a survival benefit in randomized clinical trials so far. Recently, we and others have revealed that GSCs and DGCs cooperated with each other and were involved in tumor progression and shaping an immunosuppressive tumor microenvironment.^11^ Although GSC-targeted therapies were well investigated, no therapy targeting both GSCs and DGCs has been reported. Since GSCs reversibly alter their state and show therapeutic resistance,^8^ the efficacy of therapy targeting only GSCs is limited. Therefore, we proposed dual target therapy, which significantly altered the cell cycle and induced apoptosis in both GSCs and DGCs. In this study, candidate drugs for combination therapy were determined based on publicly available gene expression and drug sensitivity data in cancer cells. We first calculated GSC and DGC signatures and then identified candidate drugs based on those data. Some reports have predicted drug susceptibility based on cellular gene expression data.^20^^,^^21^ Tang et al. predicted drug sensitivity and resistance in three-dimensional (3D) culture models by integrating the gene expression of a 3D bioprinted GBM model with drug sensitivity and gene expression data from the CCLE and CTRP.^20^ They tested the drugs predicted to be sensitive *in vitro* and reported that while the predictions and actual effects were similar, they did not match perfectly. Similarly, in our current work, there were drugs predicted to be sensitive in GSCs that showed lower IC50 values in DGCs, and vice versa. Our *in silico* screening is thus not perfect, but we consider it to be useful as a method for drug screening. In addition, this study utilized gene expression and drug sensitivity data to identify drugs effective to GSCs or DGCs. This approach could also help screen drugs that are potentially effective for malignancies other than GSCs and DGCs.

The cell cycle is a crucial pathway of cell biology and is divided into the G1, S, G2, and M phases. Cells enter the cell cycle after the transcription of released E2F in the nucleus, which is promoted by the phosphorylation of RB in the context of interaction with cyclin and CDK2 complex. Protein and lipid synthesis for mitosis and duplex DNA repair occur in the G2 phase.^22^^,^^23^^,^^24^ In GBM, dysregulations of various cellular pathways such as alterations of p53, PTEN, and the PI3K/AKT/mTOR pathway, have been reported and are involved in cell cycle abnormality.^25^ In our experimental model, we identified belinostat and dasatinib as therapeutic agents for GSCs and DGCs, respectively, both of which were previously reported to regulate the cell cycle. Belinostat induces cell-cycle arrest at the G2/M checkpoint by suppressing Cyclin B1 expression and phosphorylation of CDK1 in cancer cells, leading to apoptosis of the arrested cells.^26^^,^^27^ Dasatinib inhibits Src and the PI3K-Akt-mTOR pathway, leading to the dysregulation of cellular functions such as cell proliferation, growth, differentiation, and glucose metabolism.^28^ Others also reported that dasatinib induced cell-cycle arrest at the G1/S transition.^29^^,^^30^^,^^31^ Consistent with previous literature, belinostat monotherapy mainly suppressed the expression of genes regulating the cell cycle, especially in the G2/M transition, whereas dasatinib monotherapy suppressed not only the G2/M transition but also the PI3K-AKT-mTOR pathway, which is downstream of Src targeted by dasatinib. Combination therapy enhanced G2 arrest in GSC and the induction of apoptotic cells. Immunohistochemistry of tumor-bearing mice also showed decreased Ki-67 index and higher cleaved caspase-3 expression in the combination group. In addition to the increase in cleaved caspase-3, which is downstream of the caspase cascade, we also confirmed an increase in the expression of cleaved PARP. Both of these factors are necessary for the activation of the apoptosis pathway, and these results show that the apoptosis pathway was activated by the combination treatment.^32^ HDACi, including belinostat, have been reported to induce autophagy in addition to the cell-cycle arrest and apoptosis. Autophagy is induced by the downregulation of the PI3K-Akt-mTOR pathway. This report also states that HDACi can induce cell death by inducing autophagy without inducing apoptosis, and that it is effective even in cancer cells that show resistance to apoptosis.^33^

Dasatinib is being investigated in combination with other agents in clinical trials for the treatment of GBM; however, dasatinib showed no survival benefit.^15^^,^^34^ HDACis are also being clinically investigated, but their efficacy as monotherapy is limited.^13^ In contrast, some efficacy has been achieved in combination with other therapeutic agents.^35^ While there are no reports showing that dasatinib and belinostat alone are effective as new treatment options, the potential therapeutic effects of combining the two drugs have not yet been investigated. Blood–brain barrier penetration of dasatinib was evaluated in a central nervous system leukemia model created by transplanting a chronic myelogenous leukemia cell line into the brains of mice. In this report, the brains of mice were collected after drug administration and evaluated using high-performance liquid chromatography/mass spectroscopy (HPLC/MS).^36^ The results suggested that dasatinib has sufficient central nervous system penetration to produce antitumor activity. Belinostat has also been reported as a drug that penetrates the blood–brain barrier, and its use in combination with current standard treatments is being investigated.^37^ Based on these observations, we consider that both dasatinib and belinostat have blood–brain barrier permeability and have the possibility to be used clinically for GBM. In this report, we focused on the G2/M checkpoint and PI3K-Akt-mTOR pathway based on RNA-seq results. These pathways were not only suppressed in the control group compared to the single agent group, but were also suppressed in the monotherapy group compared to the combination group. In addition to the pathways focused on in this study, other pathways that may affect the antitumor effect were also suppressed, indicating the need for further investigation. Even if monotherapy is not sufficiently effective, the combination of dasatinib and belinostat may have therapeutic effects due to its mechanism of action that targets both GSCs and DGCs.

In this study, we used GSC and DGC gene expression signatures to apply *in silico* screening to select candidate drugs targeting GSCs or DGCs and identified belinostat for GSCs and dasatinib for DGCs. Combination therapy with belinostat and dasatinib successfully showed significant improvement of efficacy *in vitro* and *in vivo*. These results suggest that the dual targeting of GSCs and DGCs could be a therapeutic strategy for overcoming intratumoral heterogeneity.

### Limitations of the study

We predicted treatment responses of GSCs and DGCs based on gene expression data and demonstrated the potential of expression-guided drug selection. Although overall trends were captured, discrepancies remained between predicted and observed responses, indicating limitations of bulk transcriptomic data. To enhance prediction accuracy, the integration of additional bulk and single-cell transcriptomic datasets is being considered.

Combination effects were evaluated only *in vivo*. While belinostat and dasatinib are clinically approved, their combination has not been studied clinically. Further investigation is required to optimize the administration route, dosage, and schedule for clinical application.

## Resource availability

### Lead contact

Requests for further information and resources should be directed to and will be fulfilled by the lead contact, Yoshihiro Otani (yotani@okayama-u.ac.jp).

### Materials availability

This study did not generate new unique reagents.

### Data and code availability

• The raw RNA-sequencing data are deposited in the Gene Expression Omnibus. These data can be obtained at GEO database (dataset: GSE241247). The data analyzed in this study were obtained from Gene Expression Omnibus (GEO) at GSE54791.
• All software and packages used in this study are publicly available and are listed in the key resources table and the STAR Methods section. This article does not include any newly generated code.
• Additional information necessary to reanalyze the data reported in this article is available upon request from lead contact.

## Acknowledgments

This study was supported by grants-in-aid for Scientific Research from the Japanese Ministry of Education, Culture, Sports, Science, and Technology (No. 22K16660 to AU, No. 21K20803 and 22K16687 to YO, No. 21K16632 and No. 24K12285 to KF, and No. 23H03017 to ST), the Takeda Science Foundation (to AU), and the Japan Brain Foundation (to AU).

## Author contributions

S.H., A.U., and Y.O. contributed to the study conception and design. S.H., Y.S., R.I., M.H. T.U., R.I, S.N., N.T., K.M., N.K., Y.K., J.I., and K.F. performed the experiments. Data analysis was performed by S.H. and A.U. The first draft of the article was written by S.H., A.U., and Y.O., and all authors reviewed on previous versions of the article. Y.M., Y.T., Y.S., H.W., S.T., and I.D. provided scientific input for the article. All authors read and approved the final article.

## Declaration of interests

The authors declare no competing interests.

## STAR★Methods

### Key resources table

**Table:** 

REAGENT or RESOURCE	SOURCE	IDENTIFIER
**Antibodies**		

CD 133 mouse	BioLegend,	Cat# 372808; RRID: AB_2650667
Phosphor-cdc2 rabbit	Cell Signaling Technology	Cat# 4539; RRID: AB_560953
Cyclin B1 rabbit	Cell Signaling Technology	Cat# 12231; RRID: AB_2783553
Phospho- Akt rabbit	Cell Signaling Technology	Cat# 4060; RRID: AB_2315049
Akt rabbit	Cell Signaling Technology	Cat# 9272; RRID: AB_329827
Cleaved-PARP rabbit	Cell Signaling Technology	Cat# 5625; RRID: AB_10699459
PARP rabbit	Cell Signaling Technology	Cat# 9542; RRID: AB_2160739
α-Tubulin rabbit	Cell Signaling Technology	Cat# 2144; RRID: AB_2210548
HLA mouse	Abcam	Cat# ab70328; RRID: AB_1269092
Cleaved Caspase3 rabbit	Cell Signaling Technology	Cat# 9661S; RRID: AB_2341188
Ki-67 mouse	Cell Signaling Technology	Cat# 9449S; RRID: AB_2797703
Mouse IgG	Cell Signaling Technology	Cat# 7076; RRID: AB_330924
Rabbit IgG	Cell Signaling Technology	Cat# 7074; RRID: AB_2099233

**Chemicals, peptides, and recombinant proteins**		

Neurobasal medium	Invitrogen	Cat# 21103049
L-glutamine	FUJIFILM wako	Cat# 073-05391
B27 supplement	Thermo Fisher Scientific	Cat# 17504044
N2 supplement	Thermo Fisher Scientific	Cat# 10502048
0.5× penicillin G/Streptmycin sulate/amphotericin B complex	FUJIFILM Wako	Cat# 161-23181
Astrocyte Growth Medium	Cell Applications	Cat# 882AK-05f
Recombinant human EGF	Peprotech	Cat# AF-100-15
Recombinant human FGF	Peprotech	Cat# AF100-18B
D-MEM/Ham’s F-12	FUJIFILM Wako	Cat# 048-29785
FBS	Sigma	Cat# F7524
GSK-J4	Abcam 144395	Cat# 144395
SR8278	Abcam 146173	Cat# 146173
Belinostat	TCI chemicals	Cat# 866323
Dasatinib	Abcam	Cat# 142050
Lovastatin	Abcam	Cat# 120614
Fluvastatin	Sigma Aldrich	Cat# SML0038

**Critical commercial assays**		

Cell Titer 96	Promega	Cat# G5421
Annexin V-FITC Apoptosis Detection Kit	Nacalai Tesque	Cat# 15342-54

**Deposited data**		

RNA sequencing dataset from three matched pairs (MGG4, 6, and 8) of GSCs and DGCs	Suva et al.^16^	GEO: GSE54791
RNA sequencing dataset from MGG8 treated with dasatinib, belinostat and combination therapy	This study	GEO: GSE241247

**Experimental models: Cell lines**		

GBM12	Mayo Clinic	N/A
Normal human astrocytes	Cell applications	Cat#882AK-05f
MGG4	Massachusetts General Hospital	N/A
MGG8	Massachusetts General Hospital	N/A

**Experimental models: Organisms/strains**		

BALB/c-nu/nu	CLEA Japan	–

**Software and algorithms**		

GraphPad Prism 9	GraphPad Software	https://www.graphpad.com/
SynergyFinder 2.0	Ianevski et al.^38^	https://synergyfinder.fimm.fi
Gene Set Enrichment Analysis	Subramanian et al.^39^	http://software.broadinstitute.org/gsea/msigdb/annotate.jsp
Cancer Cell Line Encyclopedia	Rees et al.^40^	https://sites.broadinstitute.org/ccle/
Cancer Therapeutics Response Portal	Seashore-Ludlow et al.^41^	https://portals.broadinstitute.org/ctrp/
Fast QC	Andrews et al.^42^	http://www.bioinformatics.babraham.ac.uk/projects/fastqc/
Trimmomatic	Bolger et al.^43^	http://www.usadellab.org/cms/index.php?page=trimmomatic
Kallisto	Bray et al.^44^	http://pachterlab.github.io/kallisto/
Image Lab Software	Bio-Rad	N/A
ImageJ	NIH	N/A
MACSQuantify Software	Miltenyi Biotec	N/A
BZ-X analyzer	KEYENCE	N/A
Single Sample Gene Set Enrichment Analysis	Barbie et al.^45^	https://www.genepattern.org/modules/docs/ssGSEAProjection/5/

### Experimental model and study participant details

#### Public data acquisition

A deposited RNA sequencing dataset from three matched pairs (MGG4, 6, and 8) of GSCs and DGCs (GSE54791) were downloaded from the NCBI Gene Expression Omnibus (GEO) database.^16^

#### Cell culture

Patient-derived GBM primary cultures MGG4 and MGG8 were established as previously described.^46^^,^^47^ Patient-derived primary GBM culture GBM12 was obtained from Dr. Jann Sarkaria’s lab (Mayo Clinic). GSCs and DGCs were isolated by fluorescence-activated cell sorting as previously described.^11^ In brief, MGG4 and MGG8 cells were labeled with CD 133 Antibody (BioLegend, San Diego, CA, US, #372808). Then, the cells were incubated with propidium iodide. CD133-positive and CD133-negative cells were sorted by a BD FACSAria Ⅲ cell sorter (Becton Dickinson, Franklin Lakes, NJ, US). The CD133-positive cells were cultured as GSCs and CD133-negative cells were cultured as DGCs in the following medium. GSCs were cultured in neurobasal medium (Invitrogen, Waltham, MA, US) supplemented with 3 mmol/L l-Glutamine (FUJIFILM Wako, Osaka, Japan), 1× B27 supplement without vitamin A (Thermo Fisher Scientific, Waltham, MA, US), 0.5× N2 supplement (Thermo Fisher Scientific), 20 ng/mL recombinant human EGF (Peprotech, East Windsor, NJ, USA), 20 ng/mL recombinant human FGF2 (Peprotech), and 0.5× penicillin G/streptomycin sulfate/amphotericin B complex (FUJIFILM Wako). DGCs were cultured in Dulbecco’s modified Eagle’s medium (DMEM) supplemented with 10% fetal bovine serum (FBS), 100 U/ml penicillin, and 100 μg/ml streptomycin. GSCs and DGCs established in this manner have been previously characterized by their marker expression status.^11^ Cells were maintained at 37°C and 5% CO_2_ and confirmed to be free of mycoplasma.

Normal human astrocytes (NHAs) were purchased from Cell Applications, Inc. (San Diego, CA, US). NHAs were cultured in Astrocyte Growth Medium (Cell Applications, Inc.). Cultures were maintained in a humidified atmosphere of 5% CO_2_ at 37°C.

#### Mouse

All animal experiments were conducted with approval from the Committee on the Ethics of Animal Experimentation at Okayama University. For intracranial tumor xenograft models, female BALB/c-nu/nu mice (6 weeks old) were purchased from CLEA Japan Inc. (Fujinomiya, Japan).

### Method details

#### Drug sensitivity prediction

We analyzed a deposited RNA sequencing dataset from three matched pairs of GSCs and DGCs (GSE54791). On the basis of the ranking metric score (signal-to-noise ratio), GSC and DGC signature genes, differentially expressed between GSCs and DGCs, were defined.

Drug sensitivity and gene expression data were retrieved from the Cancer Therapeutics Response Portal (CTRP) (https://portals.broadinstitute.org/ctrp/).^40^^,^^41^^,^^48^ The Single Sample Gene Set Enrichment Analysis Projection (ssGSEA Projection) module on GenePattern (https://www.genepattern.org/modules/docs/ssGSEAProjection/5/) was used to calculate the GSC and DGC signature score for each cell line in the dataset obtained from the Cancer Cell Line Encyclopedia (CCLE) (https://sites.broadinstitute.org/ccle/).^39^^,^^40^^,^^45^ GSC and DGC signature scores were then correlated with area under the curve (AUC) values for drug susceptibility for each compound tested. Correlation r-value was plotted and statistical analyses were corrected for multiple test correction.

#### Cell viability assay and measurement of IC50

*In vitro* cell viability assays were performed using MGG4, MGG8, and NHAs. MGG4 and MGG8 were exposed to GSK-J4 (Abcam, Cambridge, UK, #144395), SR8278 (Abcam, #146173), belinostat (TCI Chemicals, Tokyo, Japan, #866323), dasatinib (Abcam, #142050), lovastatin (Abcam, #120614), and fluvastatin (Sigma Aldrich, St. Louis, MO, USA, #0038). MGG4 and MGG8 adjusted to 1 × 10^5^ cells/ml or NHAs adjusted to 2.5 × 10^4^ cells/ml were plated on a 96-well plate at 100 μl/well. After culturing for 24 h, a drug adjusted from 0 nM to 10,000 nM was added to each well, and cells were cultured for an additional 72 h. Cell viability was measured by Cell Titer 96 (Promega, WI, US, #G5421), and dose–response curves were created with GraphPad Prism version 9 and analyzed using nonlinear regression. The values of half-maximal inhibitory concentration (IC50) were calculated by using log (inhibitor) versus response including variable slope (four parameters) statistics and normalized in GraphPad Prism.

#### Analysis of drug combination *in vitro*

Based on the results of the IC50 of each drug as described above, the drugs for combination therapy were determined. MGG8 cells adjusted to 1 × 10^5^ cells/ml were plated on a 96-well plate at 100 ml/well and cultured for 24 h. Dasatinib (0, 0.1, 1, 10, 12.5, 25 nM for DGCs; 0, 1, 10, 25, 50, 100 nM for GSCs) and belinostat (0, 1, 10, 100, 250, 500 nM for both DGCs and GSCs) were added to each well. Seventy-two hours later, cell viability was measured by Cell Titer 96. Dose effect curves were drawn for their anti-cancer cell line effects using Prism software version 9. Choosing the %inhibition as the phenotypic response, the anti-cancer cell effect of combination therapy was analyzed using SynergyFinder 2.0 (https://synergyfinder.fimm.fi).^38^ Briefly, the mean inhibitory rates of the combination of belinostat and dasatinib at different concentrations were input, respectively. The high single agent (HSA) reference model was selected in this study. This model states that the expected combination effect is the maximum of the single drug responses at corresponding concentrations. The comprehensive synergy score and synergy maps of drug combination were obtained automatically by the software using the HSA model. The synergy score was used to evaluate the efficacy of the combination as follows: scores < −10: the interaction between two drugs was likely to be antagonistic; from −10 to 10: the interaction between two drugs was likely to be additive; and >10: the interaction between two drugs was likely to be synergistic.

#### RNA-sequence and data analysis

MGG8 cells were treated as follows: 500 nM of belinostat (belinostat group), 50 nM of dasatinib (dasatinib group), 500 nM of belinostat and 50 nM of dasatinib (combination group), or 0.1% dimethyl sulfoxide (DMSO, control group). Seventy-two hours later, the cells were harvested, and RNA was isolated using an All prep RNA/DNA mini kit (QIAGEN, Hilden, Germany) and on-column DNase (Invitrogen). Poly(A) RNA was prepared by a Poly(A) mRNA Magnetic Isolation Module (New England Biolabs, Ipswich, MA, US), and library preparation was performed with a NEBNext® Ultra™II Directional RNA Library Prep Kit for Illumina® (New England Biolabs). Sequencing was conducted using the NovaSeq 6000 system (Illumina Inc., San Diego, CA, US). The fastq files were quality checked using FastQC.^42^ Adapter sequences were removed using Trimmomatic.^43^ Quantification of transcript abundance was performed using Kallisto.^44^ Identification of differentially expressed genes (DEGs) was performed as follows. First, the z-score was calculated based on the TPM of samples prepared with three samples for each group. Second, we compared the expression difference between the control group and the treatment group (belinostat, dasatinib, combination) by multiple unpaired *t*-test with the z-score calculated above. Finally, genes with FC > 2 or FC < 0.5 and false discovery rate (FDR < 0.05) were defined as DEG. Gene set enrichment analysis (GSEA) was performed using the online GSEA web portal (http://software.broadinstitute.org/gsea/msigdb/annotate.jsp) and the GSEA desktop application.^39^ All data were deposited in the Gene Expression Omnibus (accession number GSE241247).

#### Western blot analysis

Cells were collected and lysed in ice-cold lysis buffer (20 mM Tris pH 7.5, 150 mM NaCl, 1 mM EDTA, 1 mM EGTA, 1.0% Triton X-100, 1 tablet/10 cc buffer of PhosSTOP, and protease inhibitor cocktail). After sonication, lysates were centrifuged at 15,000 rpm at 4°C for 10 min. Protein concentration of the supernatants was measured using a Qubit Protein Assay Kit (Invitrogen, #Q33211). The supernatants were added to a one-third volume of 4× SDS sample buffer (240 mM Tris–HCl, pH 6.8, 8% SDS, 40% glycerol, 0.1% bromophenol blue, and 20% 2-mercaptoethanol) and boiled at 100°C for 5 min. Fifteen microliters of protein samples were applied to Mini-PROTEAN TGX Precast Gels (Bio-Rad Laboratories, Hercules, CA, USA, #456-1035-B03) and then transferred to an Immun-Blot PVDF membrane for protein blotting (Bio-Rad Laboratories, #162-0177). The membrane was blocked with 5% skim milk. After blocking, the membranes were incubated overnight at 4°C with the following primary antibodies: anti-phospho-cdc2 (1:1000, Cell Signaling Technology, Danvers, MA, US, #4539), anti-Cyclin B1 (1:1000, Cell Signaling Technology, #12231), anti-phospho-Akt (Ser 473) (1:1000, Cell Signaling Technology, #4060), anti-Akt (1:1000, Cell Signaling Technology, #9272), anti-Cleaved PARP (1:1000, Cell Signaling Technology, #5625), anti-PARP (1:1000, Cell Signaling Technology, #9542), and anti-α-Tubulin (1:1000, Cell Signaling Technology, #2144). The secondary antibodies were horseradish peroxidase–conjugated anti-mouse IgG (1:5,000, Cell Signaling Technology, #7076) and anti-rabbit IgG (1:5,000, Cell Signaling Technology, #7074). HRP signals were visualized using the ECL® Prime Western Blotting Detection systems (GE Healthcare, Little Chalfont, UK) and analyzed using the Molecular imager® ChemiDoc™ XRS+ with image Lab™ software (Bio-Rad Laboratories). For quantitative evaluation, each protein was verified three times.

#### Cell cycle analysis

Cells were treated with 0.1% DMSO (control group), belinostat (500 nM, belinostat group), dasatinib (50 nM, dasatinib group), or combination (500 nM of belinostat and 50 nM of dasatinib, combination group) and incubated at 37°C for 24 h. Cells were fixed in cold 70% ethanol and incubated in PBS containing propidium iodide (PI, 50 ug/ml) and RNase-A (100 ug/ml) at 4°C for 4 h. Flow cytometry analysis was performed in MACSQuant (Miltenyi Biotec, Bergisch Gladbach, Germany) at the Central Research Laboratory, Okayama University Medical School. All samples were passed through a 40 mm cell strainer before analysis.

#### Apoptosis detection

A total of 1 × 10^5^ tumor cells were treated with 0.1% DMSO (control group), belinostat (500 nM, belinostat group), dasatinib (50 nM, dasatinib group), or combination (500 nM of belinostat and 50 nM of dasatinib, combination group) and incubated at 37°C for 24 h. Apoptosis assay was performed using an Annexin V-FITC Apoptosis Detection Kit (Nacalai Tesque, Kyoto, Japan, #15342-54). Cells were collected and stained with Annexin V-FITC and PI according to the manufacturer’s protocol. All samples were passed through a 40 mm cell strainer and analysis was performed using MACSQuant. The percentage of Annexin V- FITC single-positive cells showing early apoptosis and Annexin V- FITC and PI double-positive cells showing late apoptosis were measured as apoptotic cells.

#### Mouse intracranial tumor model

All animal experiments were performed with approval from the Committee on the Ethics of Animal Experimentation at Okayama University (OKU-2021784). The intracranial tumor xenograft murine models were established as previously described.^11^^,^^49^^,^^50^ Briefly, the mice were anesthetized, and tumor cells (2 × 10^5^ cells) were stereotactically injected into the right frontal lobe. The injection point was 3 mm lateral and 1 mm anterior from the bregma and 3 mm deep from the dura.

To evaluate the effect of drug treatment *in vivo*, mice bearing intracranial tumors were treated with vehicle (5% DMSO, control group), belinostat (250 mg/kg, belinostat group), dasatinib (25 mg/kg, dasatinib group), or a combination of both drugs (combination group). Dosages of dasatinib and belinostat were determined with reference to the papers reported to date, which showed a certain antitumor effect and did not cause death due to adverse events.^29^^,^^51^^,^^52^ For the MGG8 intracranial tumor mice model, both agents were administered by oral gavage every 24 h for 5 days per cycle from day 38 to 42 and 45 to 49 after tumor implantation, with a 2-day rest period given between the two cycles. Tissue was harvested on day 50 after transplantation at the end of the second treatment cycle. For the GBM12 intracranial tumor mice model, both agents were administered by oral gavage every 24 h for 5 days per cycle from day 28 to 32 after tumor implantation. Tissue was harvested on day 33 after implantation.

#### Immunohistochemistry

Immunohistochemistry was performed as previously described.^49^ Sections were stained with primary antibodies such as anti-HLA Class I (0.05 mg/ml, Abcam, #ab70328), anti-Cleaved-Caspase3 (1: 400, Cell Signaling Technology, #9661S), and anti-Ki-67 (1: 1000, Cell Signaling Technology, #9449S) overnight at 4°C, and detected using an HRP-conjugated compact polymer system (Envision+ system- HRP labelled Polymer anti-Rabbit (Dako, Glostrup, Denmark, K4003) and anti-Mouse (Dako, K4001). DAB was used as the chromogen. The sections were then counterstained with hematoxylin. Images were acquired using a BX-50 microscope (Olympus, Tokyo, Japan). To evaluate the positivity rates of cleaved caspase-3 and Ki-67, four experimental groups (control group, dasatinib group, belinostat group, and combination group) were prepared, with three mice per group. Immunohistochemical staining was performed on tumor tissues collected from each mouse, and the positive rate was calculated for three sites per mouse, with the average serving as the result for that mouse. The positive rate for each group was calculated by averaging the positive rates of the three mice in that group. All evaluation was performed at a magnification of 400×.

#### Magnetic resonance imaging (MRI) and tumor volume measurement

MRI was conducted with the approval of the animal study protocol (OKU-2021590). Intracranial tumor model mice were generally anesthetized with isoflurane and given a gadolinium contrast medium intraperitoneally (Gadovist, Bayer, Japan). The mice were screened with high-resolution axial T2-weighted images using a Rapid Acquisition with Relaxation Enhancement (RARE) sequence to evaluate brain tumor size and to monitor its evolution stage, using repetition time (TR)/effective echo time (TE) = 1200/8 ms. MRI data were acquired and processed by using ParaVision 5.1 software (Bruker BioSpec 4.7 T, Ettlingen, Germany).

Tumor volume was calculated using the following formula using ImageJ. Sum of each tumor area per each slice (mm^2^) × 0.5 (500 μm/section). Tumor volume was measured in three mice per group.

### Quantification and statistical analysis

GraphPad Prism 9 software was used to conduct statistical analysis of all data. Data were represented as the mean and standard error of the mean (SEM). The Student’s *t*-test was used for comparisons between two groups. Comparisons between multiple groups were performed with one-way analysis of variance (ANOVA) with Tukey’s multiple comparisons test. Pearson’s correlation test was used to measure the strength of the association between two variables. The chi-squared test was performed to examine differences between categorical variables. *p* values were designated as ∗*p* < 0.05, ∗∗*p* < 0.01 ∗∗∗*p* < 0.001, ∗∗∗∗*p* < 0.0001, and ns: non-significant (*p* > 0.05). Details of exact p-values and statistical analyses are provided in the supplementary Excel file (Table S7).

## Supplemental information

Document S1. Figures S1–S5 and Tables S1 and S2

Table S3. Signature scores of differentiated glioblastoma cells and glioblastoma stem cells in cancer cell lines

Table S4. Area under the curve values obtained from cancer therapeutics response portal

Table S5. Correlation between differentiated glioblastoma cell signature scores and area under the curve values

Table S6. Correlation between glioblastoma stem cell signature scores and area under the curve values

Table S7. Details of quantification and statistical analysis

Data S1. Raw images of Western Blot

## References

[1] Stupp R., Mason W.P., van den Bent M.J., Weller M., Fisher B., Taphoorn M.J.B., Belanger K., Brandes A.A., Marosi C., Bogdahn U. (2005). Radiotherapy plus concomitant and adjuvant temozolomide for glioblastoma. N. Engl. J. Med..

[2] Stupp R., Taillibert S., Kanner A., Read W., Steinberg D., Lhermitte B., Toms S., Idbaih A., Ahluwalia M.S., Fink K. (2017). Effect of Tumor-Treating Fields Plus Maintenance Temozolomide vs Maintenance Temozolomide Alone on Survival in Patients With Glioblastoma: A Randomized Clinical Trial. JAMA.

[3] Reardon D.A., Brandes A.A., Omuro A., Mulholland P., Lim M., Wick A., Baehring J., Ahluwalia M.S., Roth P., Bähr O. (2020). Effect of Nivolumab vs Bevacizumab in Patients With Recurrent Glioblastoma: The CheckMate 143 Phase 3 Randomized Clinical Trial. JAMA Oncol..

[4] Otani Y., Yoo J.Y., Shimizu T., Kurozumi K., Date I., Kaur B. (2022). Implications of immune cells in oncolytic herpes simplex virotherapy for glioma. Brain Tumor Pathol..

[5] Weller M., Butowski N., Tran D.D., Recht L.D., Lim M., Hirte H., Ashby L., Mechtler L., Goldlust S.A., Iwamoto F. (2017). Rindopepimut with temozolomide for patients with newly diagnosed, EGFRvIII-expressing glioblastoma (ACT IV): a randomised, double-blind, international phase 3 trial. Lancet Oncol..

[6] Wang Q., Hu B., Hu X., Kim H., Squatrito M., Scarpace L., deCarvalho A.C., Lyu S., Li P., Li Y. (2017). Tumor Evolution of Glioma-Intrinsic Gene Expression Subtypes Associates with Immunological Changes in the Microenvironment. Cancer Cell.

[7] Gimple R.C., Yang K., Halbert M.E., Agnihotri S., Rich J.N. (2022). Brain cancer stem cells: resilience through adaptive plasticity and hierarchical heterogeneity. Nat. Rev. Cancer.

[8] Liau B.B., Sievers C., Donohue L.K., Gillespie S.M., Flavahan W.A., Miller T.E., Venteicher A.S., Hebert C.H., Carey C.D., Rodig S.J. (2017). Adaptive Chromatin Remodeling Drives Glioblastoma Stem Cell Plasticity and Drug Tolerance. Cell Stem Cell.

[9] Li D., Zhang Q., Li L., Chen K., Yang J., Dixit D., Gimple R.C., Ci S., Lu C., Hu L. (2022). β2-Microglobulin Maintains Glioblastoma Stem Cells and Induces M2-like Polarization of Tumor-Associated Macrophages. Cancer Res..

[10] Wang X., Prager B.C., Wu Q., Kim L.J.Y., Gimple R.C., Shi Y., Yang K., Morton A.R., Zhou W., Zhu Z. (2018). Reciprocal Signaling between Glioblastoma Stem Cells and Differentiated Tumor Cells Promotes Malignant Progression. Cell Stem Cell.

[11] Uneda A., Kurozumi K., Fujimura A., Fujii K., Ishida J., Shimazu Y., Otani Y., Tomita Y., Hattori Y., Matsumoto Y. (2021). Differentiated glioblastoma cells accelerate tumor progression by shaping the tumor microenvironment via CCN1-mediated macrophage infiltration. Acta Neuropathol. Commun..

[12] Hegi M.E., Diserens A.C., Gorlia T., Hamou M.F., de Tribolet N., Weller M., Kros J.M., Hainfellner J.A., Mason W., Mariani L. (2005). MGMT gene silencing and benefit from temozolomide in glioblastoma. N. Engl. J. Med..

[13] Galanis E., Anderson S.K., Miller C.R., Sarkaria J.N., Jaeckle K., Buckner J.C., Ligon K.L., Ballman K.V., Moore D.F., Nebozhyn M. (2018). Phase I/II trial of vorinostat combined with temozolomide and radiation therapy for newly diagnosed glioblastoma: results of Alliance N0874/ABTC 02. Neuro Oncol..

[14] Galanis E., Anderson S.K., Twohy E.L., Carrero X.W., Dixon J.G., Tran D.D., Jeyapalan S.A., Anderson D.M., Kaufmann T.J., Feathers R.W. (2019). A phase 1 and randomized, placebo-controlled phase 2 trial of bevacizumab plus dasatinib in patients with recurrent glioblastoma: Alliance/North Central Cancer Treatment Group N0872. Cancer.

[15] Lassman A.B., Pugh S.L., Gilbert M.R., Aldape K.D., Geinoz S., Beumer J.H., Christner S.M., Komaki R., DeAngelis L.M., Gaur R. (2015). Phase 2 trial of dasatinib in target-selected patients with recurrent glioblastoma (RTOG 0627). Neuro Oncol..

[16] Suva M.L., Rheinbay E., Gillespie S.M., Patel A.P., Wakimoto H., Rabkin S.D., Riggi N., Chi A.S., Cahill D.P., Nahed B.V. (2014). Reconstructing and reprogramming the tumor-propagating potential of glioblastoma stem-like cells. Cell.

[17] Yang L., Shi P., Zhao G., Xu J., Peng W., Zhang J., Zhang G., Wang X., Dong Z., Chen F., Cui H. (2020). Targeting cancer stem cell pathways for cancer therapy. Signal Transduct. Target. Ther..

[18] Saito N., Fu J., Zheng S., Yao J., Wang S., Liu D.D., Yuan Y., Sulman E.P., Lang F.F., Colman H. (2014). A high Notch pathway activation predicts response to gamma secretase inhibitors in proneural subtype of glioma tumor-initiating cells. Stem Cells.

[19] Otani Y., Yoo J.Y., Chao S., Liu J., Jaime-Ramirez A.C., Lee T.J., Hurwitz B., Yan Y., Dai H., Glorioso J.C. (2020). Oncolytic HSV-Infected Glioma Cells Activate NOTCH in Adjacent Tumor Cells Sensitizing Tumors to Gamma Secretase Inhibition. Clin. Cancer Res..

[20] Tang M., Xie Q., Gimple R.C., Zhong Z., Tam T., Tian J., Kidwell R.L., Wu Q., Prager B.C., Qiu Z. (2020). Three-dimensional bioprinted glioblastoma microenvironments model cellular dependencies and immune interactions. Cell Res..

[21] Basu A., Bodycombe N.E., Cheah J.H., Price E.V., Liu K., Schaefer G.I., Ebright R.Y., Stewart M.L., Ito D., Wang S. (2013). An Interactive Resource to Identify Cancer Genetic and Lineage Dependencies Targeted by Small Molecules. Cell.

[22] Wang Z. (2021). Regulation of Cell Cycle Progression by Growth Factor-Induced Cell Signaling. Cells.

[23] Suski J.M., Braun M., Strmiska V., Sicinski P. (2021). Targeting cell-cycle machinery in cancer. Cancer Cell.

[24] Segeren H.A., van Rijnberk L.M., Moreno E., Riemers F.M., van Liere E.A., Yuan R., Wubbolts R., de Bruin A., Westendorp B. (2020). Excessive E2F Transcription in Single Cancer Cells Precludes Transient Cell-Cycle Exit after DNA Damage. Cell Rep..

[25] Brennan C.W., Verhaak R.G.W., McKenna A., Campos B., Noushmehr H., Salama S.R., Zheng S., Chakravarty D., Sanborn J.Z., Berman S.H. (2013). The somatic genomic landscape of glioblastoma. Cell.

[26] Kusaczuk M., Krętowski R., Stypułkowska A., Cechowska-Pasko M. (2016). Molecular and cellular effects of a novel hydroxamate-based HDAC inhibitor - belinostat - in glioblastoma cell lines: a preliminary report. Invest. New Drugs.

[27] Hoffmann M.J., Meneceur S., Hommel K., Schulz W.A., Niegisch G. (2021). Downregulation of Cell Cycle and Checkpoint Genes by Class I HDAC Inhibitors Limits Synergism with G2/M Checkpoint Inhibitor MK-1775 in Bladder Cancer Cells. Genes.

[28] Zhu K., Wu Y., He P., Fan Y., Zhong X., Zheng H., Luo T. (2022). PI3K/AKT/mTOR-Targeted Therapy for Breast Cancer. Cells.

[29] Zhang M., Tian J., Wang R., Song M., Zhao R., Chen H., Liu K., Shim J.H., Zhu F., Dong Z., Lee M.H. (2020). Dasatinib Inhibits Lung Cancer Cell Growth and Patient Derived Tumor Growth in Mice by Targeting LIMK1. Front. Cell Dev. Biol..

[30] Barnum K.J., O'Connell M.J. (2014). Cell cycle regulation by checkpoints. Methods Mol. Biol..

[31] Chen B., Xu X., Luo J., Wang H., Zhou S. (2015). Rapamycin Enhances the Anti-Cancer Effect of Dasatinib by Suppressing Src/PI3K/mTOR Pathway in NSCLC Cells. PLoS One.

[32] Li R., Luo R., Luo Y., Hou Y., Wang J., Zhang Q., Chen X., Hu L., Zhou J. (2022). Biological function, mediate cell death pathway and their potential regulated mechanisms for post-mortem muscle tenderization of PARP1: A review. Front. Nutr..

[33] Singh A.K., Bishayee A., Pandey A.K. (2018). Targeting Histone Deacetylases with Natural and Synthetic Agents: An Emerging Anticancer Strategy. Nutrients.

[34] Schiff D., Sarkaria J. (2015). Dasatinib in recurrent glioblastoma: failure as a teacher. Neuro Oncol..

[35] Xu K., Ramesh K., Huang V., Gurbani S.S., Cordova J.S., Schreibmann E., Weinberg B.D., Sengupta S., Voloschin A.D., Holdhoff M. (2022). Final Report on Clinical Outcomes and Tumor Recurrence Patterns of a Pilot Study Assessing Efficacy of Belinostat (PXD-101) with Chemoradiation for Newly Diagnosed Glioblastoma. Tomography.

[36] Porkka K., Koskenvesa P., Lundán T., Rimpiläinen J., Mustjoki S., Smykla R., Wild R., Luo R., Arnan M., Brethon B. (2008). Dasatinib crosses the blood-brain barrier and is an efficient therapy for central nervous system Philadelphia chromosome–positive leukemia. Blood.

[37] Gurbani S.S., Yoon Y., Weinberg B.D., Salgado E., Press R.H., Cordova J.S., Ramesh K.K., Liang Z., Velazquez Vega J., Voloschin A. (2019). Assessing Treatment Response of Glioblastoma to an HDAC Inhibitor Using Whole-Brain Spectroscopic MRI. Tomography.

[38] Ianevski A., Giri A.K., Aittokallio T. (2020). SynergyFinder 2.0: visual analytics of multi-drug combination synergies. Nucleic Acids Res..

[39] Aravind Subramanian P.T., Mootha V.K., Mukherjee S., Ebert B.L., Gillette M.A., Paulovich A., Pomeroy S.L., Golub T.R., Lander E.S., Mesirova J.P. (2005). Gene set enrichment analysis: A knowledge-based approach for interpreting genome-wide expression profiles. Proc. Natl. Acad. Sci. USA.

[40] Rees M.G., Seashore-Ludlow B., Cheah J.H., Adams D.J., Price E.V., Gill S., Javaid S., Coletti M.E., Jones V.L., Bodycombe N.E. (2016). Correlating chemical sensitivity and basal gene expression reveals mechanism of action. Nat. Chem. Biol..

[41] Seashore-Ludlow B., Rees M.G., Cheah J.H., Cokol M., Price E.V., Coletti M.E., Jones V., Bodycombe N.E., Soule C.K., Gould J. (2015). Harnessing Connectivity in a Large-Scale Small-Molecule Sensitivity Dataset. Cancer Discov..

[42] Andrews S. (2010). FastQC: A Quality Control Tool for High Throughput Sequence Data. Cancer Discov..

[43] Bolger A.M., Lohse M., Usadel B. (2014). Trimmomatic: a flexible trimmer for Illumina sequence data. Bioinformatics.

[44] Bray N.L., Pimentel H., Melsted P., Pachter L. (2016). Near-optimal probabilistic RNA-seq quantification. Nat. Biotechnol..

[45] Barbie D.A., Tamayo P., Boehm J.S., Kim S.Y., Moody S.E., Dunn I.F., Schinzel A.C., Sandy P., Meylan E., Scholl C. (2009). Systematic RNA interference reveals that oncogenic KRAS-driven cancers require TBK1. Nature.

[46] Wakimoto H., Kesari S., Farrell C.J., Curry W.T., Zaupa C., Aghi M., Kuroda T., Stemmer-Rachamimov A., Shah K., Liu T.C. (2009). Human glioblastoma-derived cancer stem cells: establishment of invasive glioma models and treatment with oncolytic herpes simplex virus vectors. Cancer Res..

[47] Wakimoto H., Mohapatra G., Kanai R., Curry W.T., Yip S., Nitta M., Patel A.P., Barnard Z.R., Stemmer-Rachamimov A.O., Louis D.N. (2012). Maintenance of primary tumor phenotype and genotype in glioblastoma stem cells. Neuro Oncol..

[48] Basu A., Bodycombe N.E., Cheah J.H., Price E.V., Liu K., Schaefer G.I., Ebright R.Y., Stewart M.L., Ito D., Wang S. (2013). An interactive resource to identify cancer genetic and lineage dependencies targeted by small molecules. Cell.

[49] Otani Y., Ichikawa T., Kurozumi K., Inoue S., Ishida J., Oka T., Shimizu T., Tomita Y., Hattori Y., Uneda A. (2018). Fibroblast growth factor 13 regulates glioma cell invasion and is important for bevacizumab-induced glioma invasion. Oncogene.

[50] Matsumoto Y., Ichikawa T., Kurozumi K., Otani Y., Fujimura A., Fujii K., Tomita Y., Hattori Y., Uneda A., Tsuboi N. (2020). Annexin A2-STAT3-Oncostatin M receptor axis drives phenotypic and mesenchymal changes in glioblastoma. Acta Neuropathol. Commun..

[51] Avril T., Etcheverry A., Pineau R., Obacz J., Jegou G., Jouan F., Le Reste P.-J., Hatami M., Colen R.R., Carlson B.L. (2017). CD90 Expression Controls Migration and Predicts Dasatinib Response in Glioblastoma. Clin. Cancer Res..

[52] Wang Z., Sun D., Chen Y.-J., Xie X., Shi Y., Tabar V., Brennan C.W., Bale T.A., Jayewickreme C.D., Laks D.R. (2020). Cell Lineage-Based Stratification for Glioblastoma. Cancer Cell.

