# Supplementary material for: Dual molecular therapy targeting tumor cell heterogeneity improves therapeutic efficacy in glioblastoma
Source: iScience. 2025 Aug 28;28(9):113456. doi: 10.1016/j.isci.2025.113456 (PMC12496224; doi:10.1016/j.isci.2025.113456)
Supplement: Document S1. Figures S1–S5 and Tables S1 and S2 [file mmc1.pdf]

iScience, Volume *28*

Supplemental information

Dual molecular therapy targeting tumor cell heterogeneity improves therapeutic efficacy in glioblastoma

Shuichiro Hirano, Atsuhito Uneda, Yoshihiro Otani, Yasuki Suruga, Ryoji Imoto, Madoka Hokama, Tsuyoshi Umeda, Ryosuke Ikemachi, Shohei Nishigaki, Nobushige Tsuboi, Keigo Makino, Naoya Kemmotsu, Yasuhito Kegoya, Yuji Matsumoto, Yusuke Tomita, Yosuke Shimazu, Joji Ishida, Kentaro Fujii, Hiroaki Wakimoto, Shota Tanaka, and Isao Date

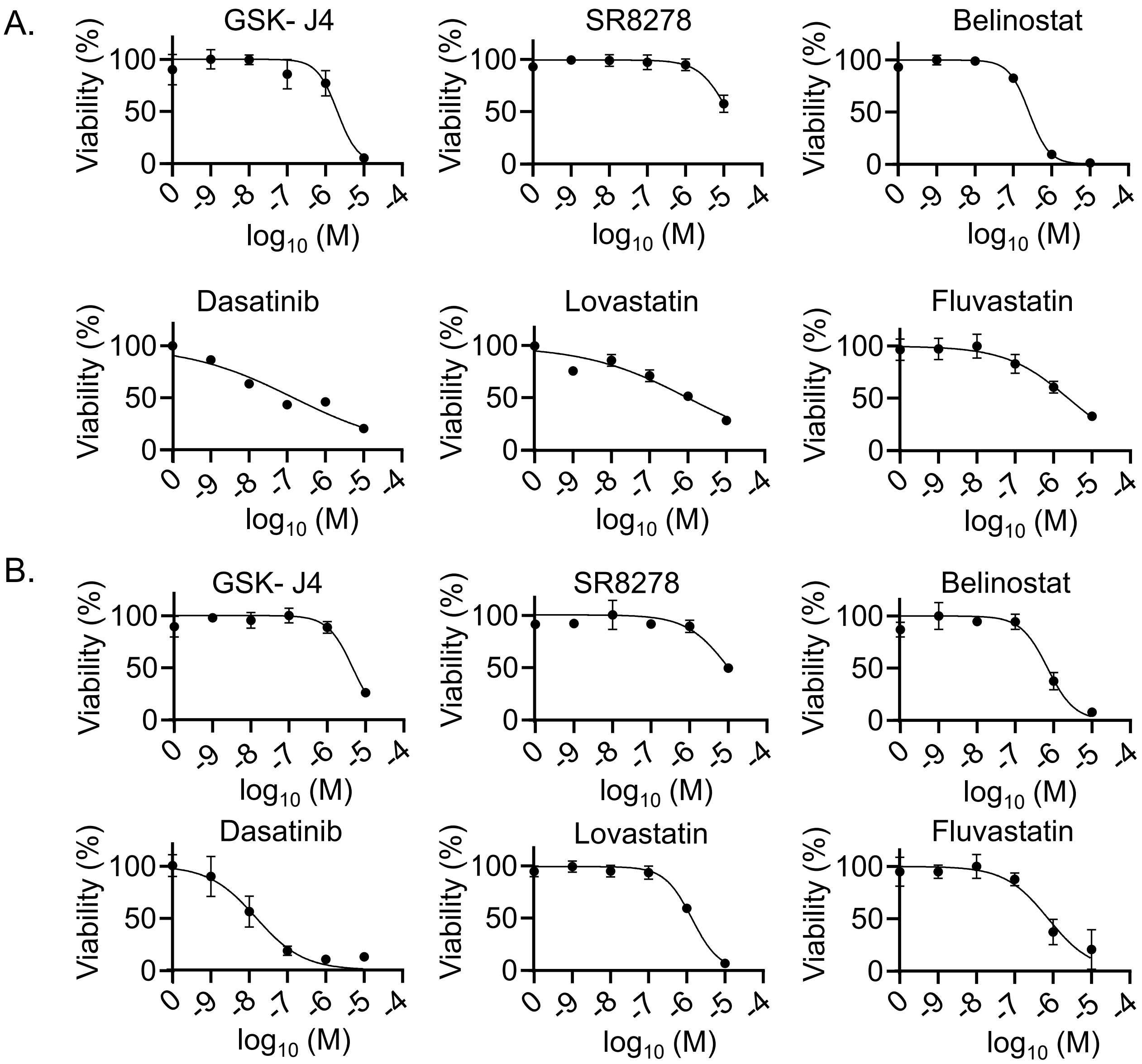

C:

Candidate drugs from GSC signature score

	MGG4 GSC	MGG4 DGC
GSK-J4	2019 nM	4,582 nM
SR8278	13,210 nM	10,060 nM
Belinostat	255.7 nM	693.1 nM

Candidate drugs from DGC signature score

	MGG4 GSC	MGG4 DGC
Dasatinib	144.5 nM	15.14 nM
Lovastatin	1,008 nM	1,357 nM
Fluvastatin	2,425 nM	743.4 nM

Figure S1: In vitro cell proliferation assay in MGG4.
A: Cell proliferation assay with GSC-targeting candidates in MGG4 GSCs (n = 5/ each drug). B: Cell proliferation assay with DGC-targeting candidates in MGG4 DGCs (n = 5/ each drug). C: Summary of IC50 for each drug.

A.

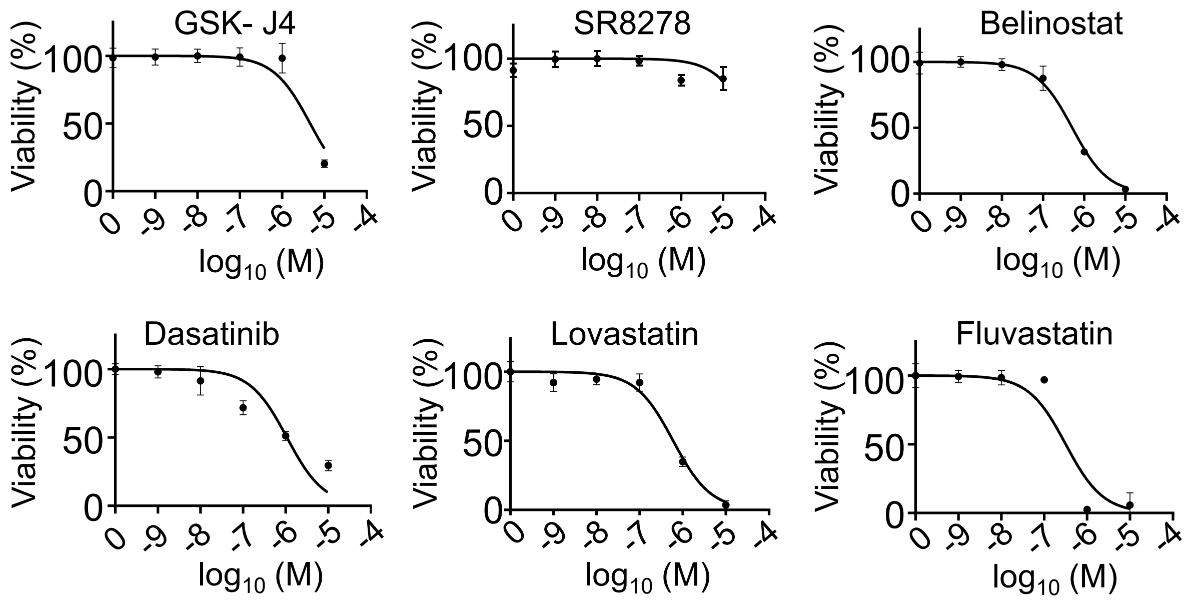

B.

Candidate drugs from GSC signature score

	NHA
GSK-J4	4588 nM
SR8278	＞10000 nM
Belinostat	510.2 nM

Candidate drugs from DGC signature score

	NHA
Dasatinib	1066 nM
Lovastatin	598.6 nM
Fluvastatin	317.1 nM

Figure S2: In vitro cell proliferation assay in NHA.
A: Cell proliferation assay with candidates in NHA (n = 5/ each drug). B: Summary of IC50 for each drug.

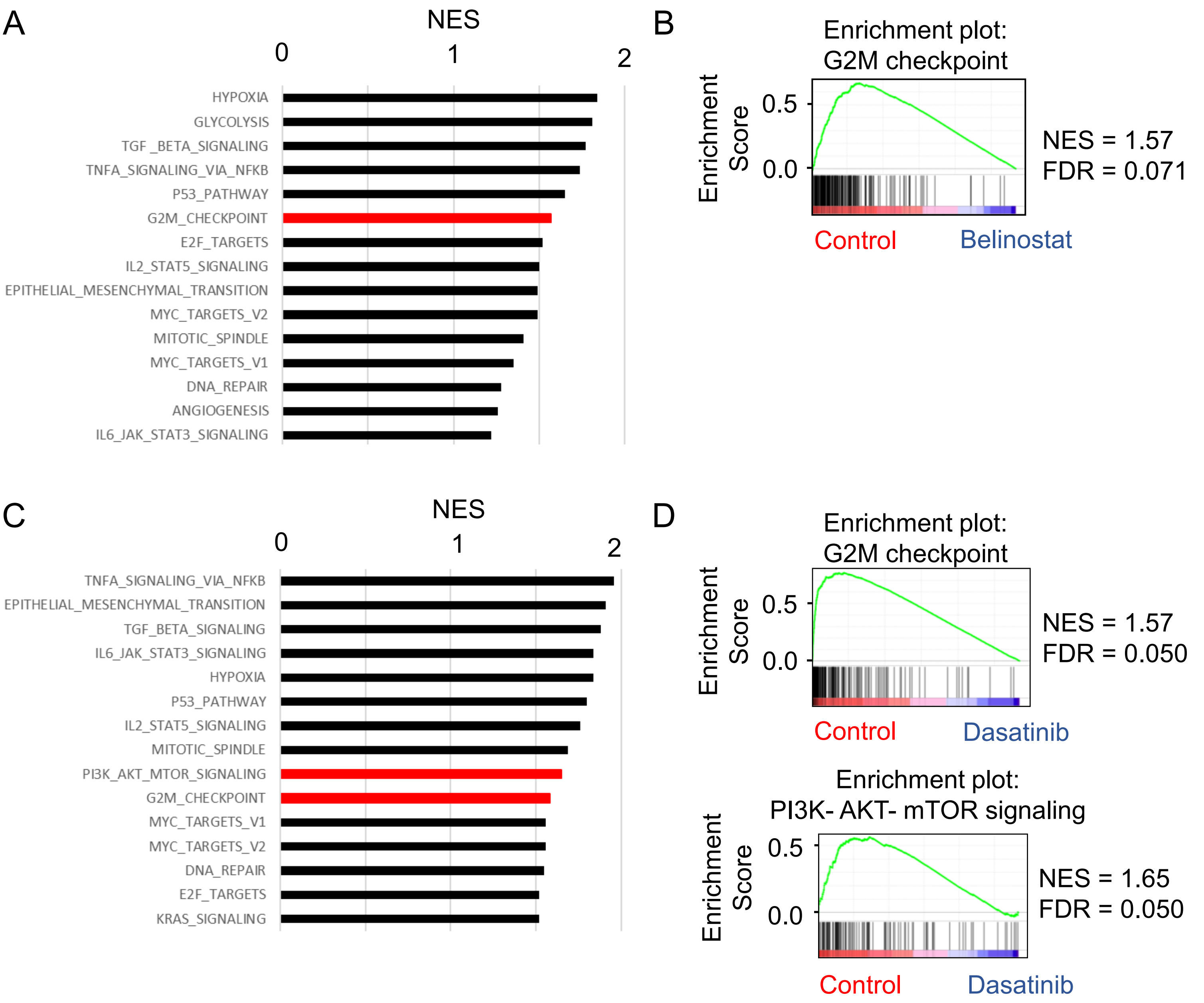

Figure S3: Hallmark gene sets downregulated in monotherapy with belinostat and dasatinib.

A: Hallmark gene sets downregulated in belinostat therapy compared with control. B: Gene set enrichment analysis showed enrichment of the G2/M transition in the control compared with belinostat therapy. C: Hallmark gene sets downregulated in dasatinib therapy compared with the control. D: Gene set enrichment analysis showed enrichment of the G2/M transition and PI3K-Akt-mTOR pathway in the control compared with dasatinib therapy.

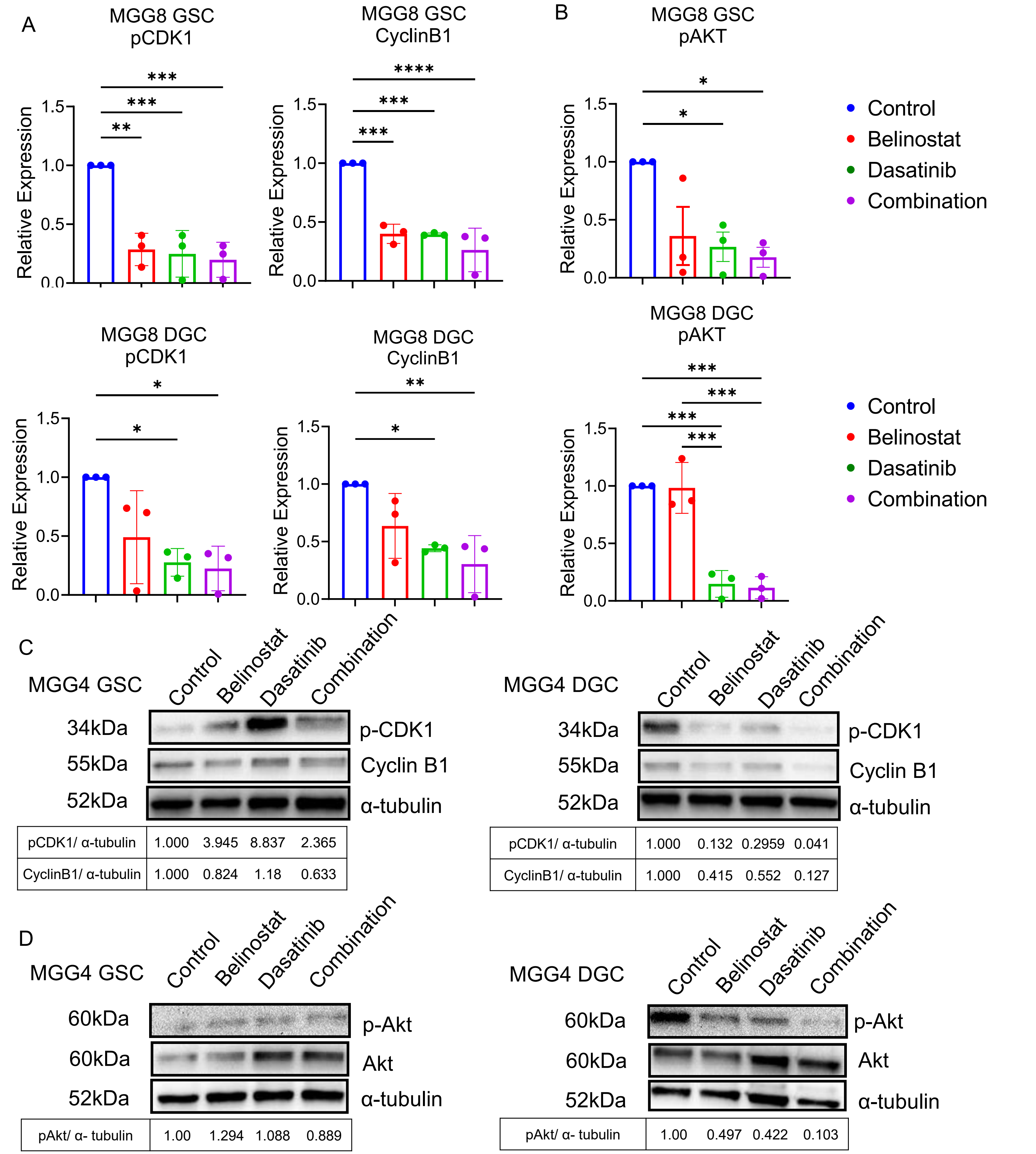

Fig. S4. Combination therapy induced G2/M arrest and PI3K-Akt-mTOR pathway inhibition

A: The relative expression of pCDK1 and Cyclin B1 in MGG8 GSCs and DGCs. B: The relative expression of pAkt in MGG8 GSCs and DGCs. C: Western blot of Cyclin B1 and p-CDK1. The relative expression values are shown below. D: Western blot of Akt and p-Akt. The relative expression values are shown below.

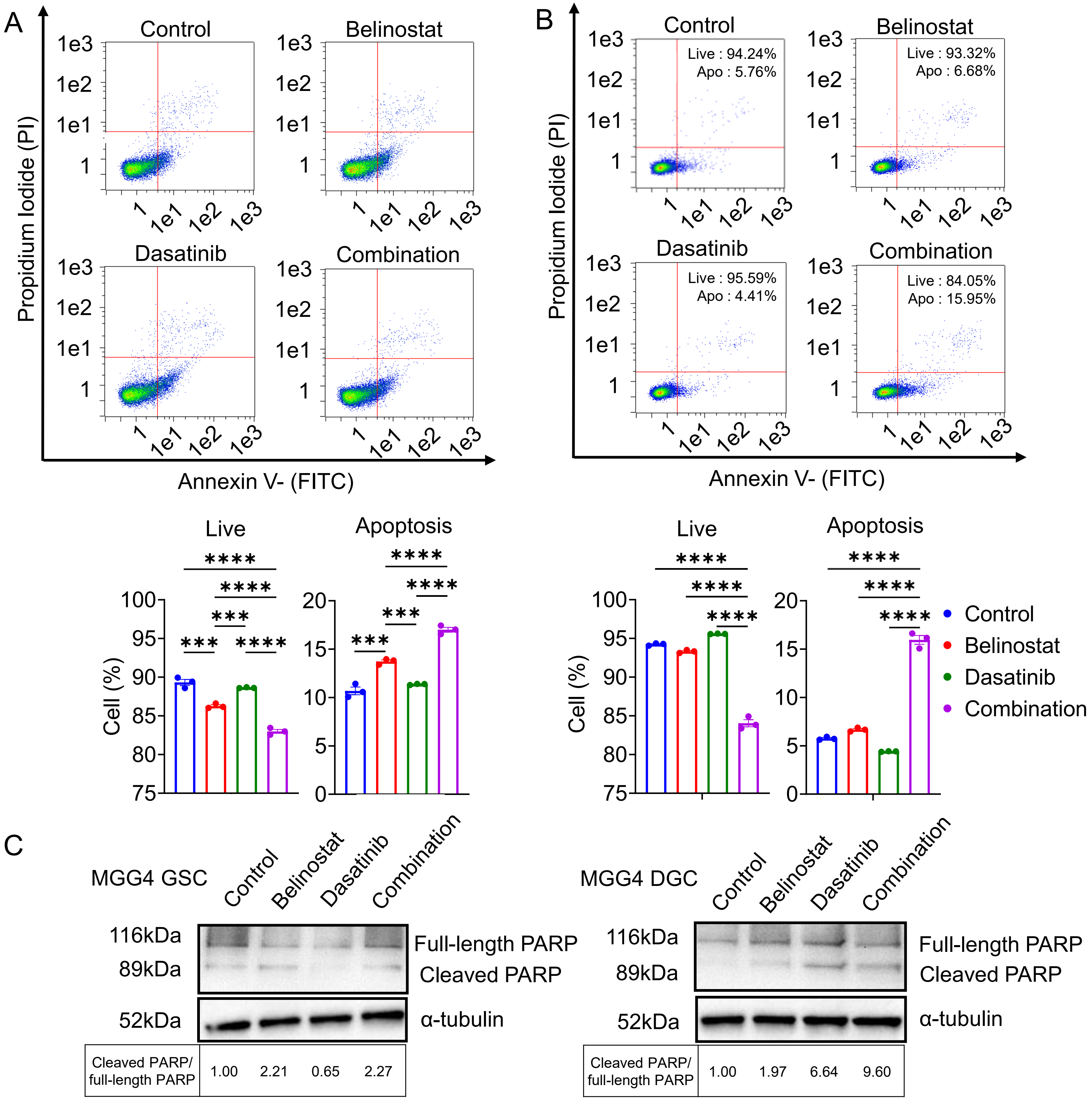

Fig. S5. Combination therapy increased apoptosis and decreased cell proliferation in glioblastoma in MGG4.
A: Apoptosis assay of MGG4 GSCs treated with vehicle (control), belinostat, dasatinib, and combination. B: Apoptosis assay of MGG4 DGCs treated with vehicle (control), belinostat, dasatinib, and combination. C: Western blot of full-length PARP and cleaved PARP in MGG4 GSCs and DGCs. Relative expression values are shown below.

Table S1. Top 50 of glioblastoma stem cells signature genes

ABAT	MYBL2
AIF1L	MYH14
ARHGDIG	NAT8L
BK250D10.8	NCAN
CADM4	NDRG2
CAMKV	NOTCH1
COL9A3	PDE3B
DHODH	PEG3
FAM198A	PREX1
FREM2	PTPRZ1
GLB1L2	RLBP1
GLDC	RNF165
GNG4	S100B
GNG7	SAPCD2
HES6	SBK1
HNRNPA3	SCARB1
IQCK	SCRG1
ISYNA1	SOX5
LAMC3	SPTBN2
LOC100499467	TEX15
LOC729080	TMPRSS5
LPHN3	TPD52
MAGED2	TRAF4
MAST1	TUBB2B
MMP15	ZNF276

Table S2. Top 50 of differentiated glioblastoma cells signature genes

AHNAK	GPR176
AHNAK2	HEBP2
ANXA2	HLX
ANXA2P1	IGFBP7
ANXA2P2	LOC100132891
ANXA2P3	LOXL2
ARHGAP24	LRP10
BEND6	LTBP2
BICC1	LY96
C10orf26	MYD88
CALCOCO2	MYH9
CASP4	MYOF
CAV2	NPC2
CD109	OBFC2A
CD24	PALLD
CLCF1	PARVA
COL5A1	PGRMC2
COL5A2	PHF11
COL6A1	POLR2B
CSRP1	PXK
CYR61	RRAS
EMP1	SPARC
FAM180A	STC2
FAP	TNFSF4
GALC	VEGFC
