# Supplementary figures and images for: Dual molecular therapy targeting tumor cell heterogeneity improves therapeutic efficacy in glioblastoma
Source: iScience. 2025 Aug 28;28(9):113456. doi: 10.1016/j.isci.2025.113456 (PMC12496224; doi:10.1016/j.isci.2025.113456)

A-tub after Akt 54.0sec

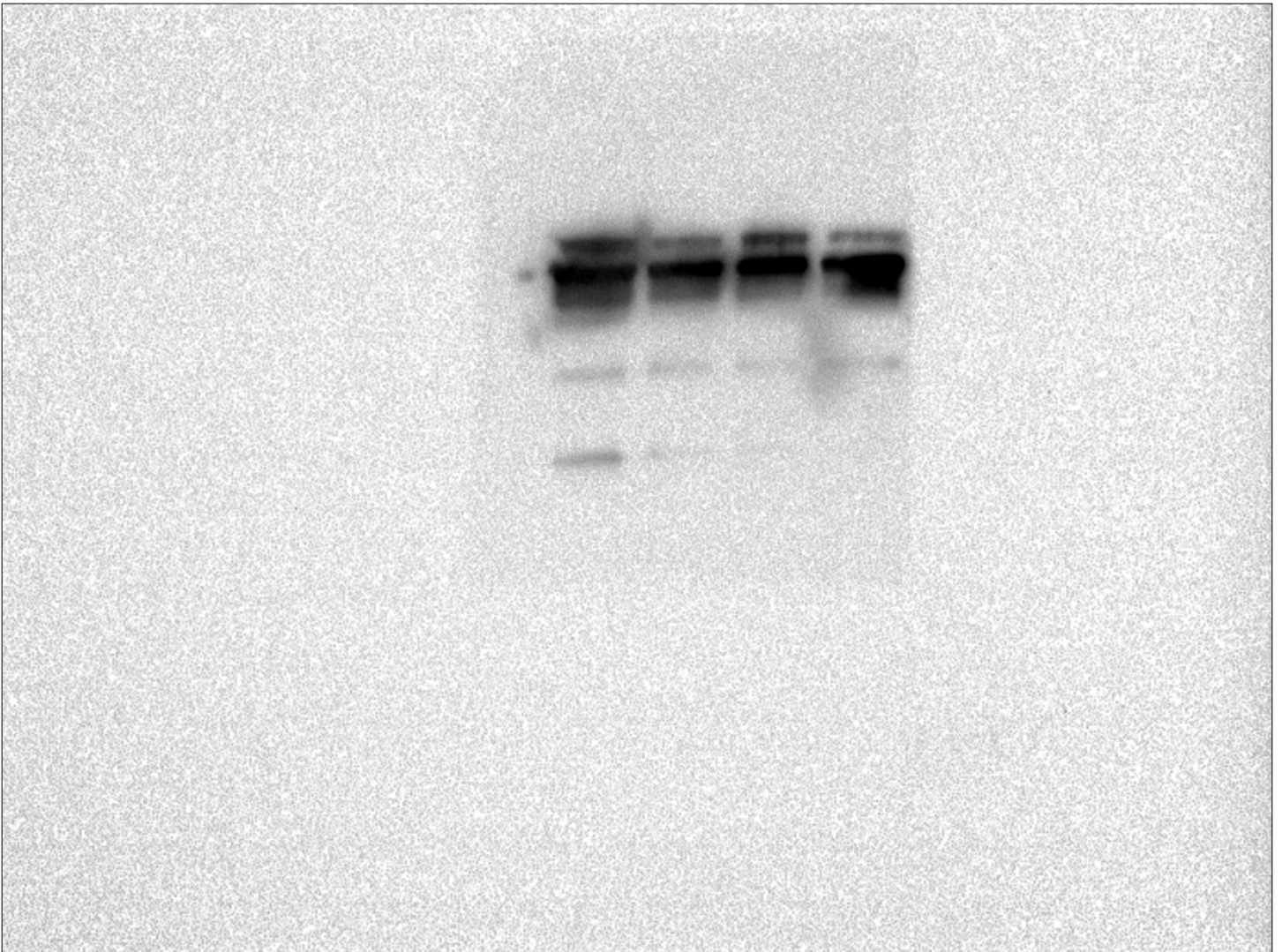

Supplement: Data S1. Raw images of Western Blot [file mmc7.zip › Western Blot/Fig3 DGC/Akt Atub DGC/A-tub.pdf]

AKt 47.8sec

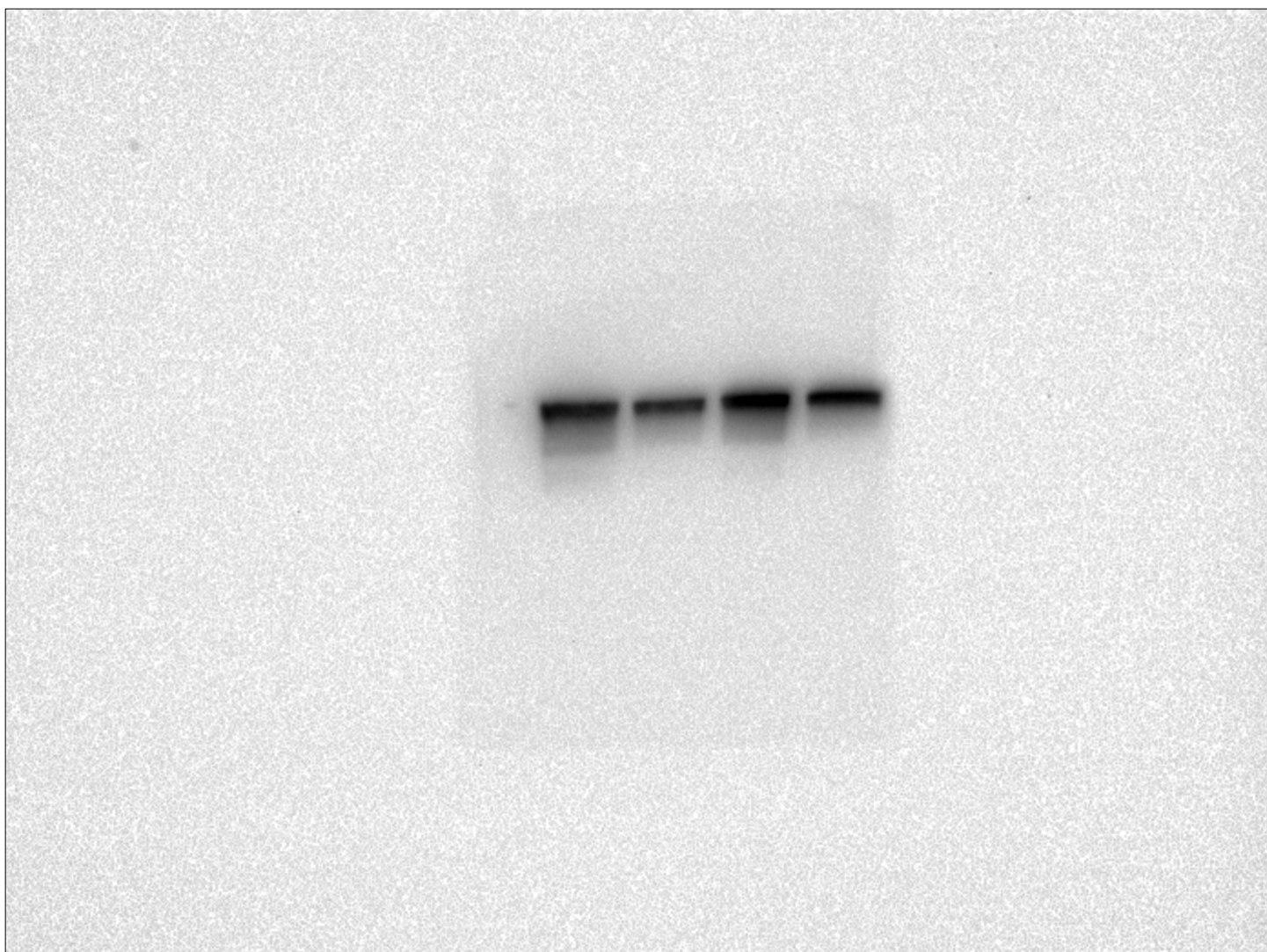

Supplement: Data S1. Raw images of Western Blot [file mmc7.zip › Western Blot/Fig3 DGC/Akt Atub DGC/Akt.pdf]

A-tub after p-AKT 60.0sec

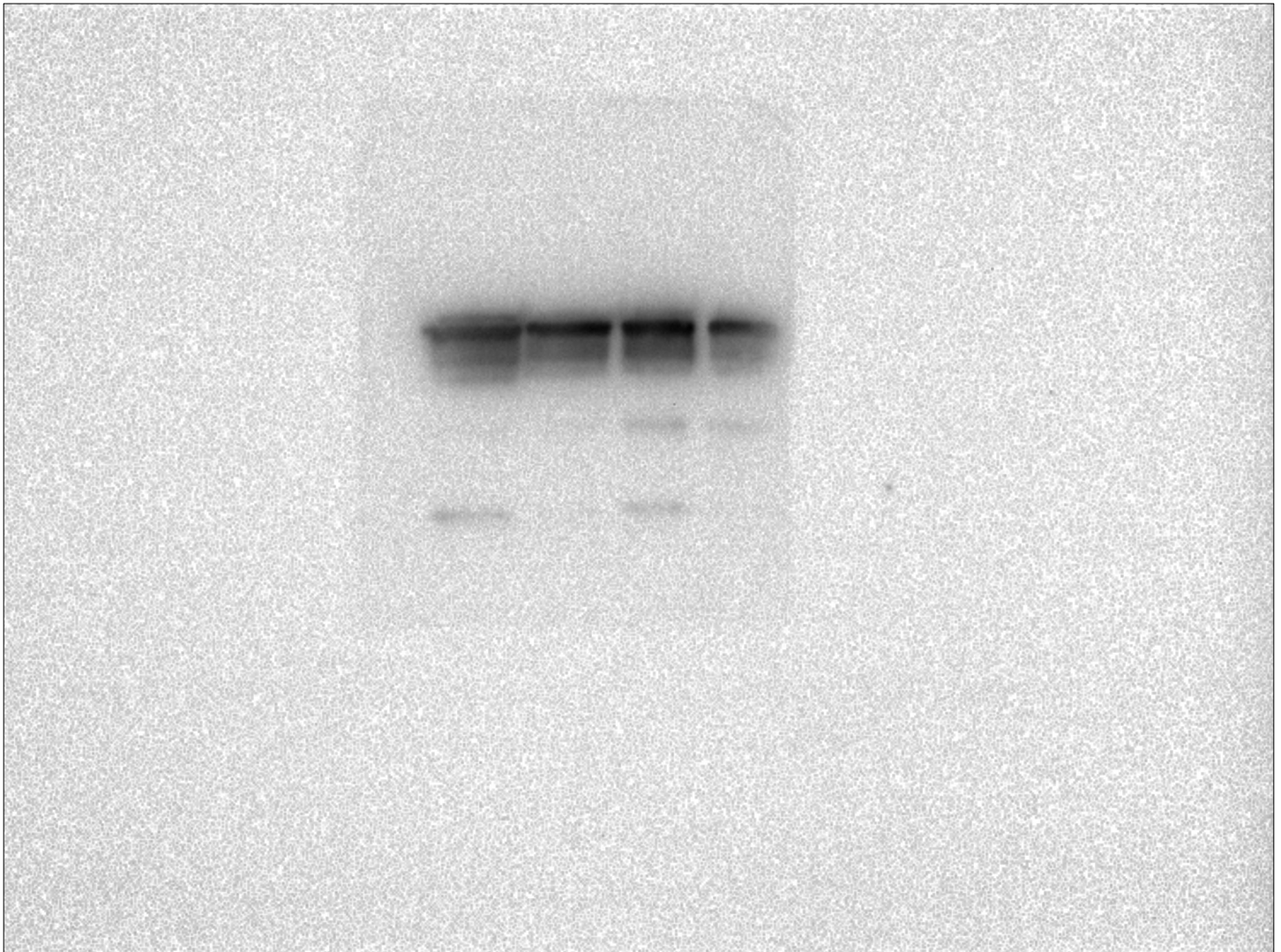

Supplement: Data S1. Raw images of Western Blot [file mmc7.zip › Western Blot/Fig3 DGC/pAkt Atub DGC/A-tub.pdf]

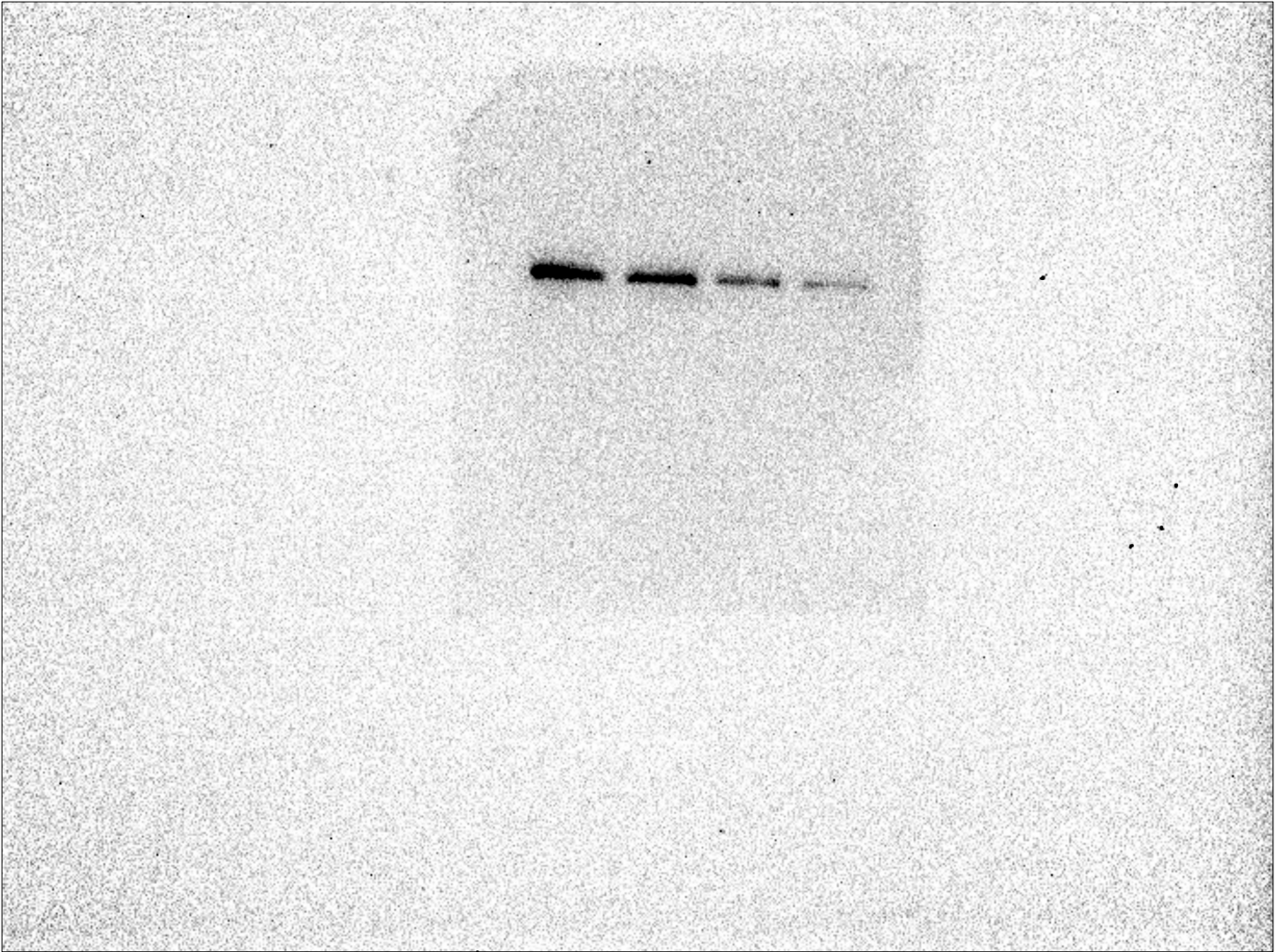

Supplement: Data S1. Raw images of Western Blot [file mmc7.zip › Western Blot/Fig3 DGC/pAkt Atub DGC/p-Akt.pdf]

A-tub after CyclinB1 p-cdc2 DGC 60sec

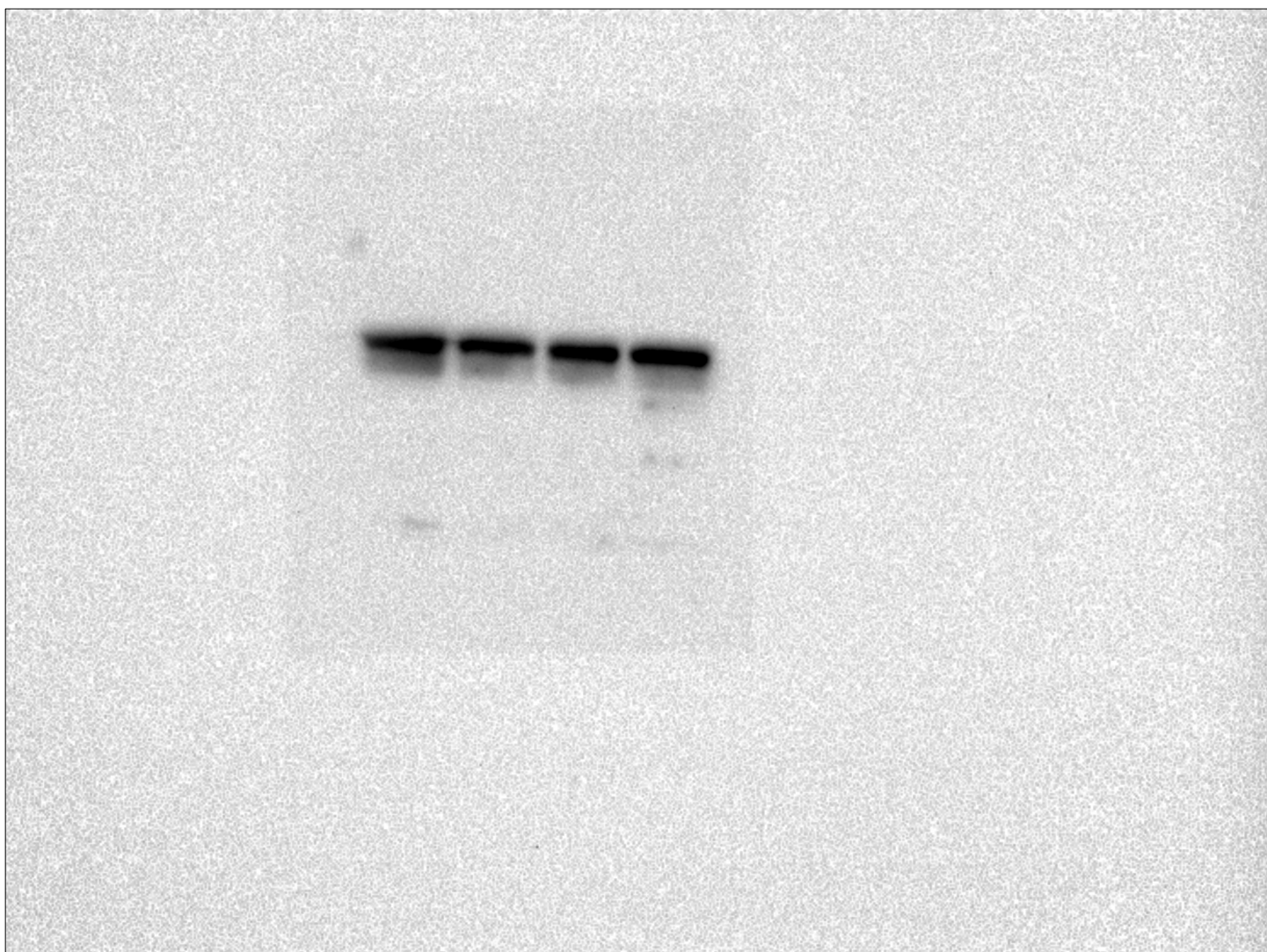

Supplement: Data S1. Raw images of Western Blot [file mmc7.zip › Western Blot/Fig3 DGC/pcdc2 cyclinB1 Atub DGC/A-tub.pdf]

CyclinB1 104.4sec

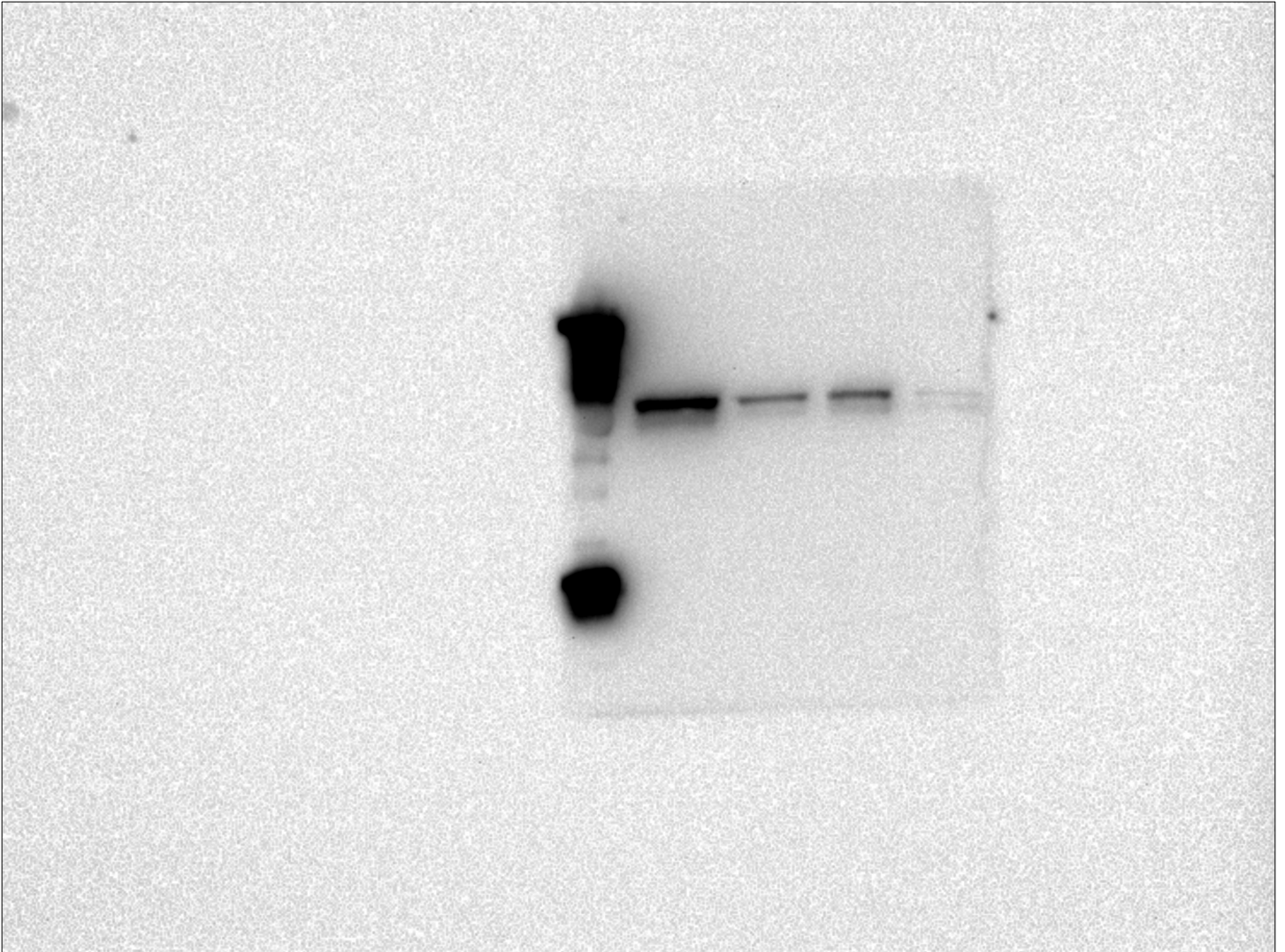

Supplement: Data S1. Raw images of Western Blot [file mmc7.zip › Western Blot/Fig3 DGC/pcdc2 cyclinB1 Atub DGC/CyclinB1.pdf]

p-cdc2 after cyclinB1 150.0sec

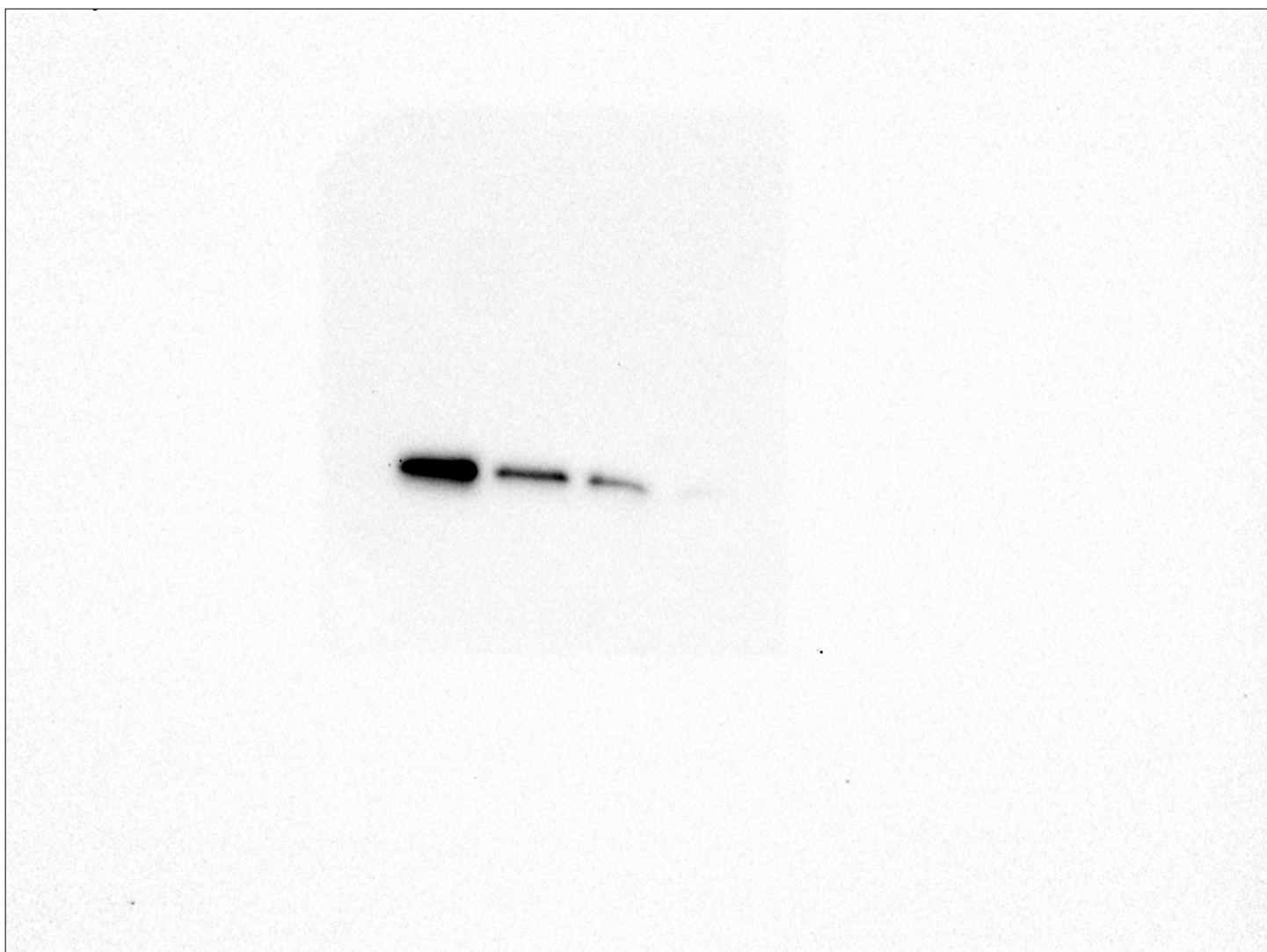

Supplement: Data S1. Raw images of Western Blot [file mmc7.zip › Western Blot/Fig3 DGC/pcdc2 cyclinB1 Atub DGC/p-cdc2.pdf]

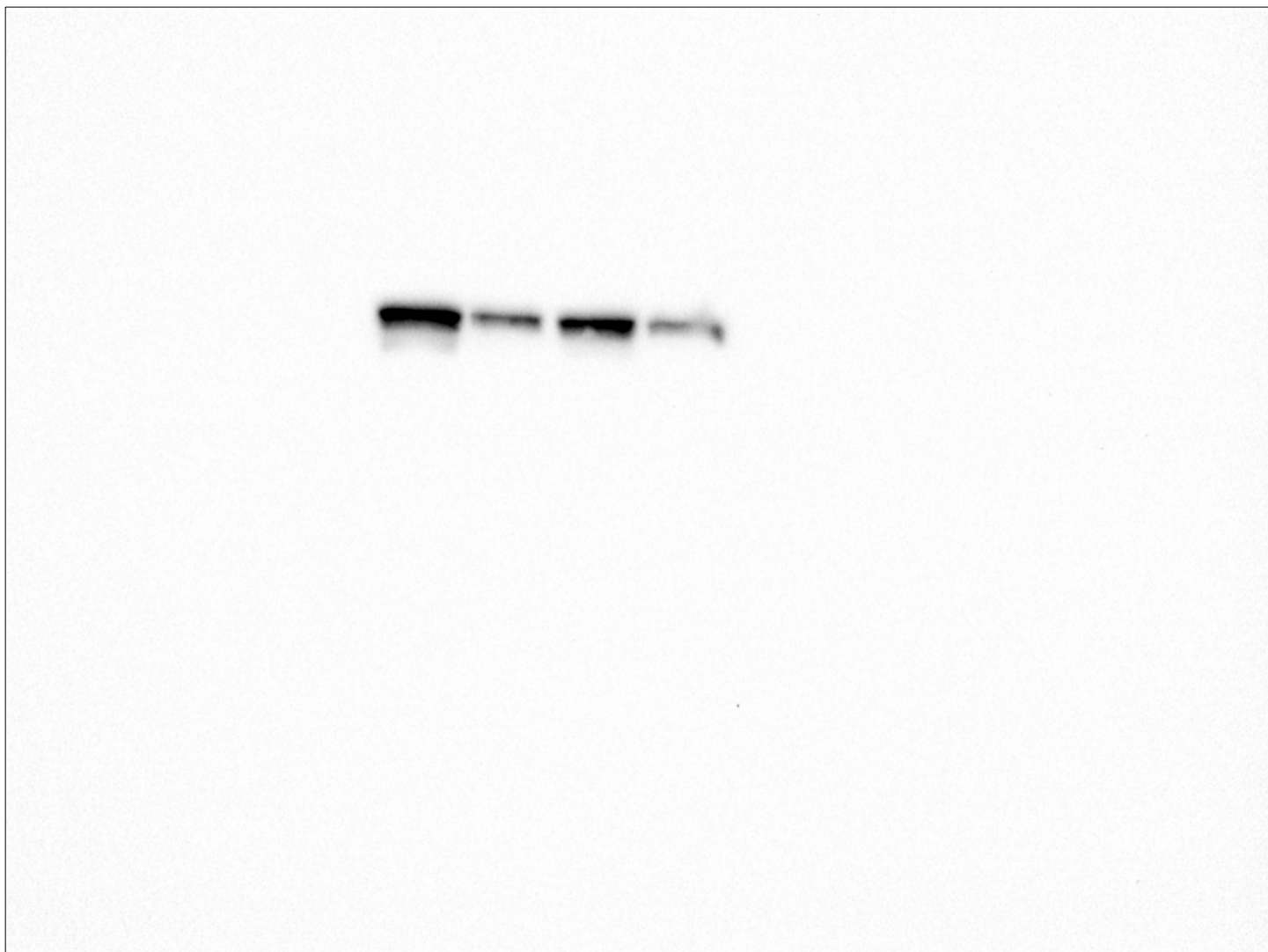

Supplement: Data S1. Raw images of Western Blot [file mmc7.zip › Western Blot/Fig3 GSC/pAkt Akt Atub GSC/akt.pdf]

Atub Akt2

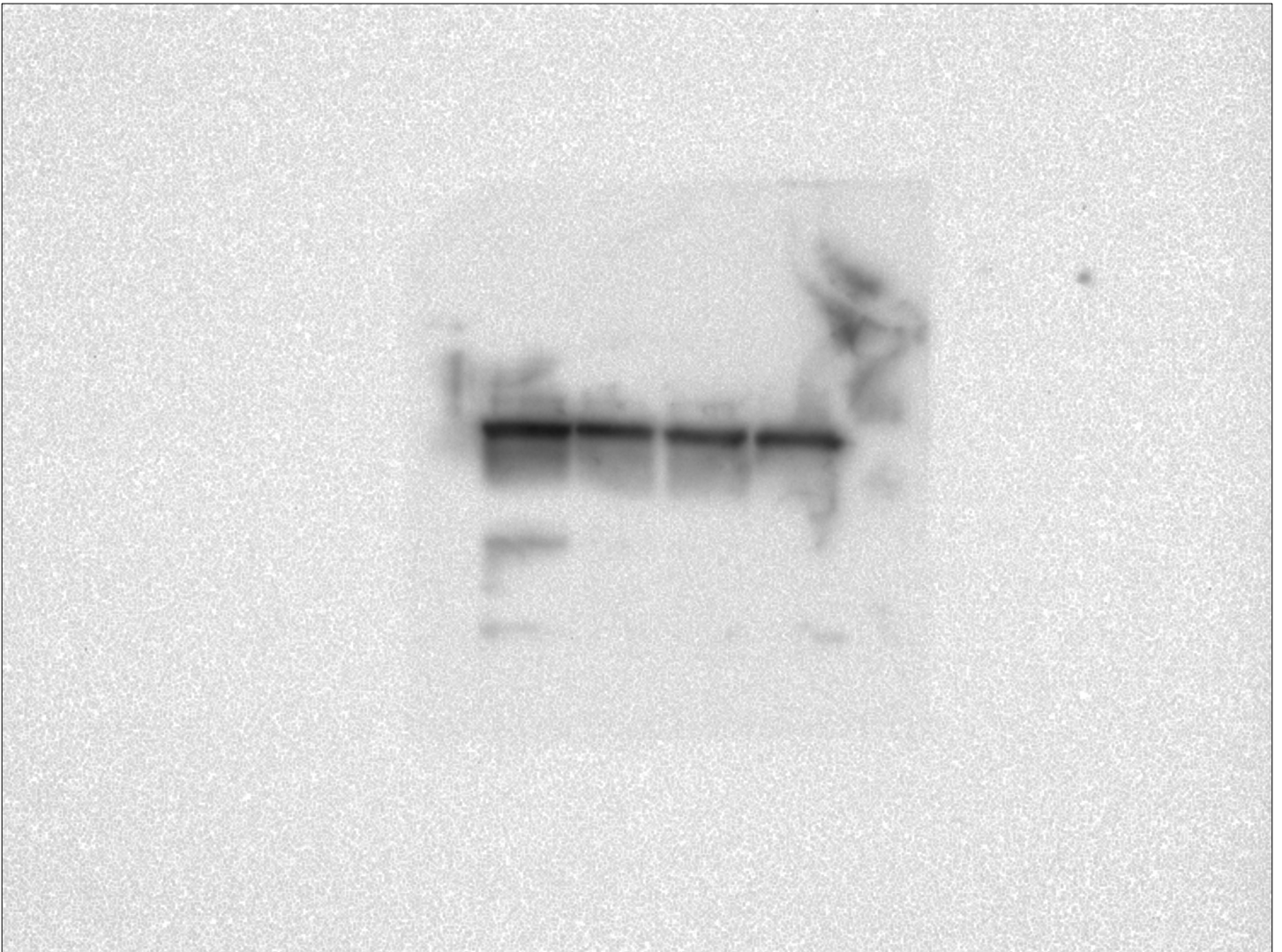

Supplement: Data S1. Raw images of Western Blot [file mmc7.zip › Western Blot/Fig3 GSC/pAkt Akt Atub GSC/Atub after Akt.pdf]

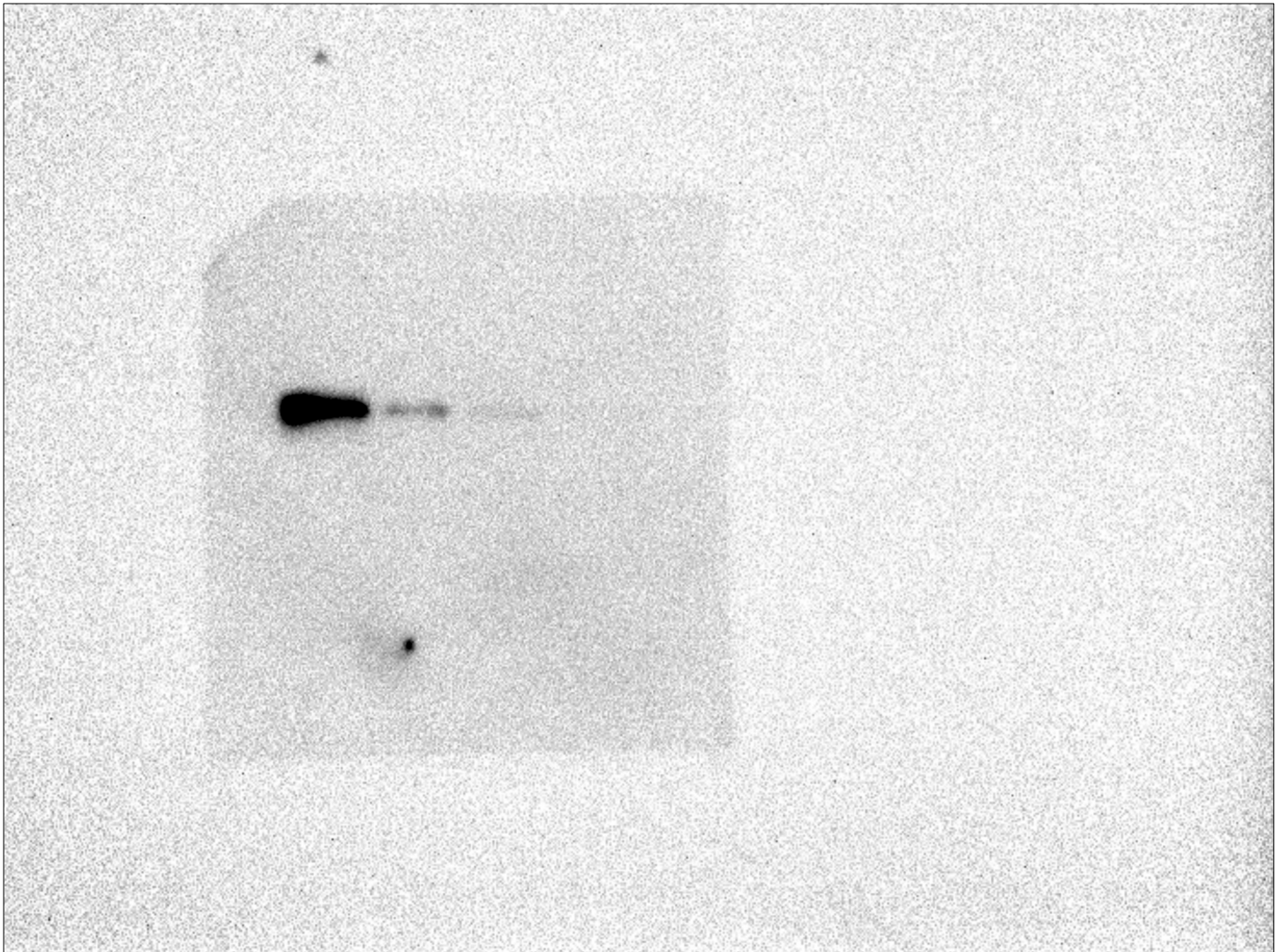

Supplement: Data S1. Raw images of Western Blot [file mmc7.zip › Western Blot/Fig3 GSC/pAkt Akt Atub GSC/p-Akt.pdf]

A tubCyclinB1

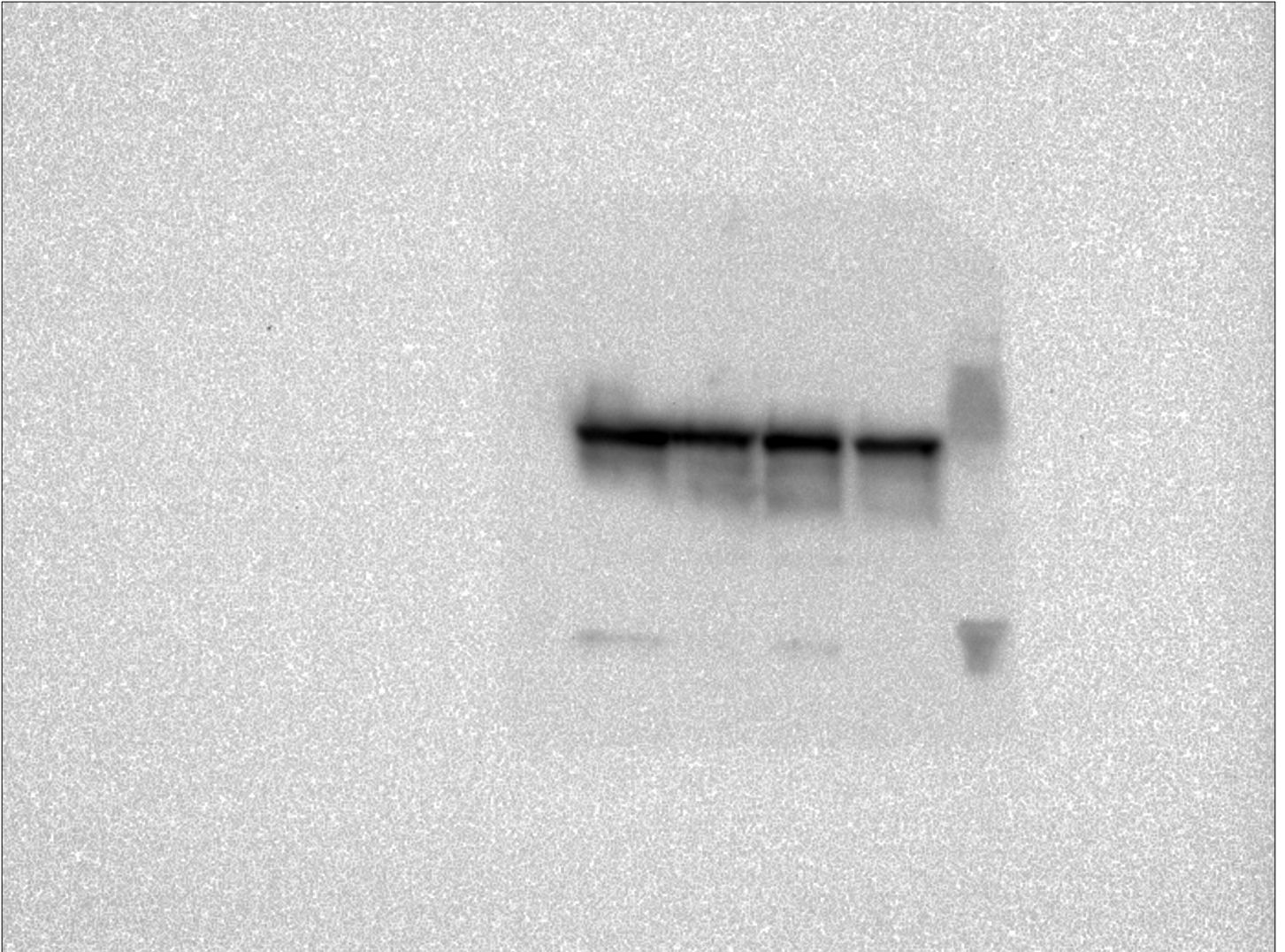

Supplement: Data S1. Raw images of Western Blot [file mmc7.zip › Western Blot/Fig3 GSC/pcdc2 CyclinB1 Atub GSC/A tub.pdf]

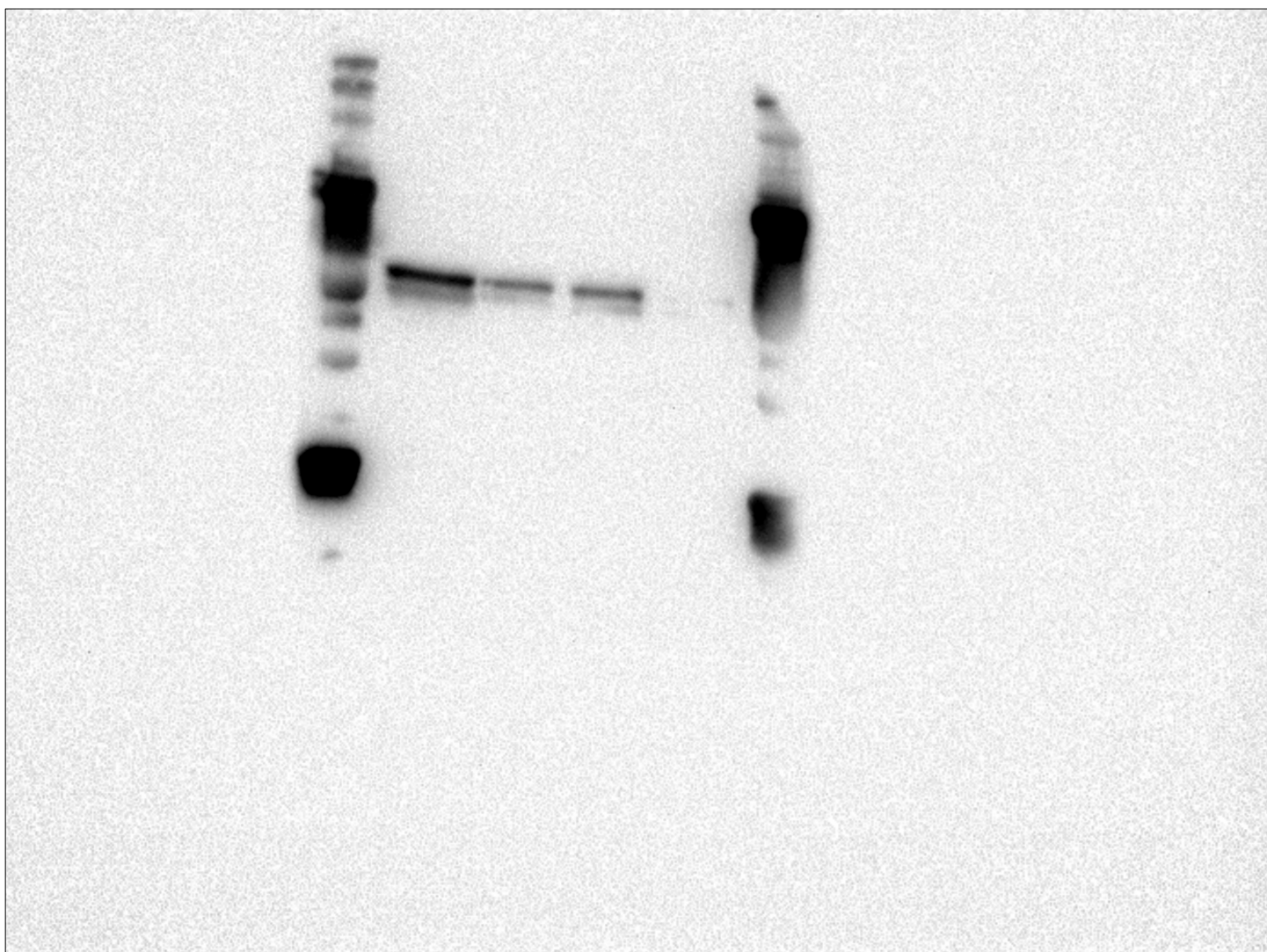

Supplement: Data S1. Raw images of Western Blot [file mmc7.zip › Western Blot/Fig3 GSC/pcdc2 CyclinB1 Atub GSC/cyclinB1 25sec.pdf]

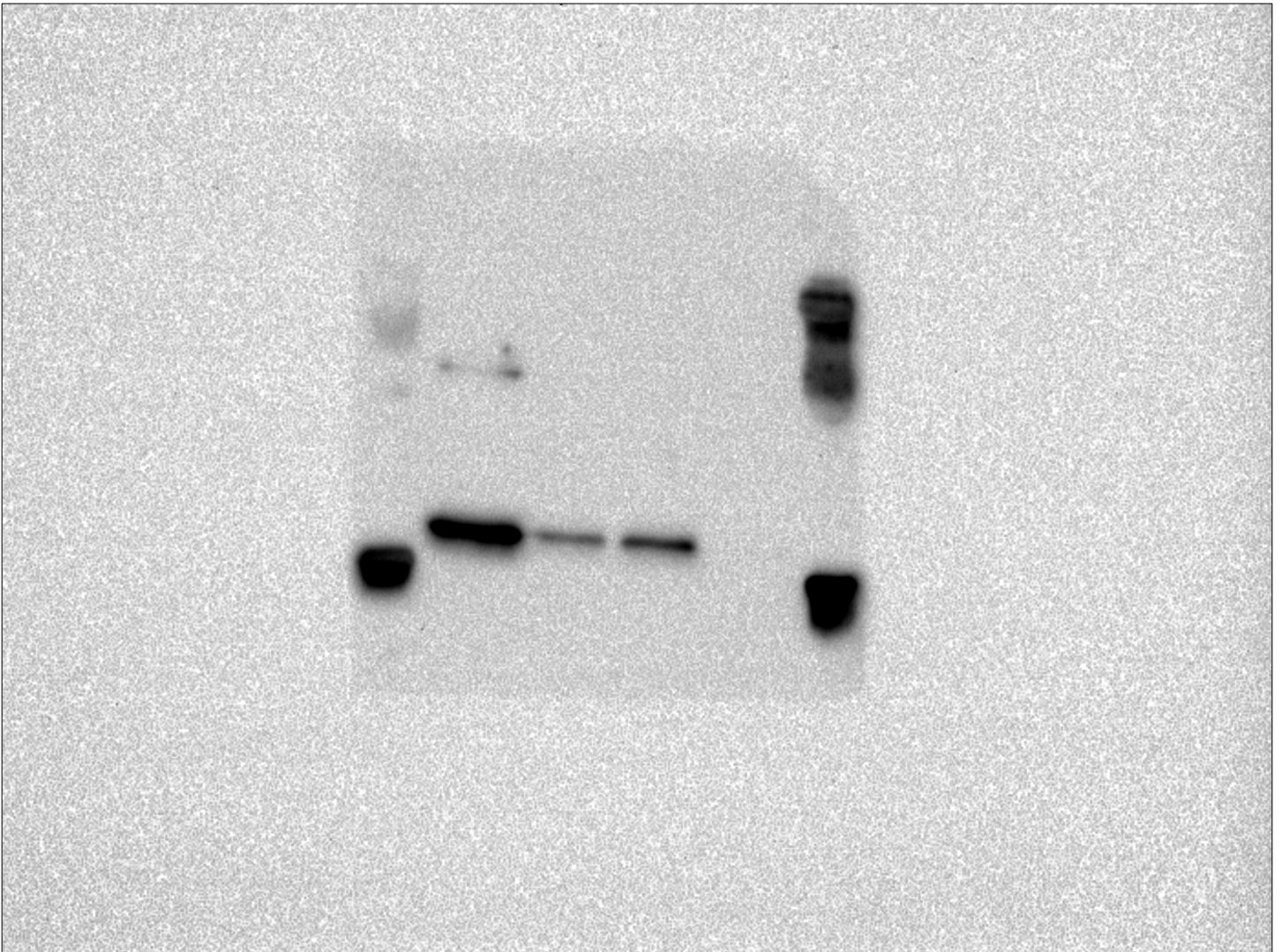

Supplement: Data S1. Raw images of Western Blot [file mmc7.zip › Western Blot/Fig3 GSC/pcdc2 CyclinB1 Atub GSC/p-cdc2 155sec 2.pdf]

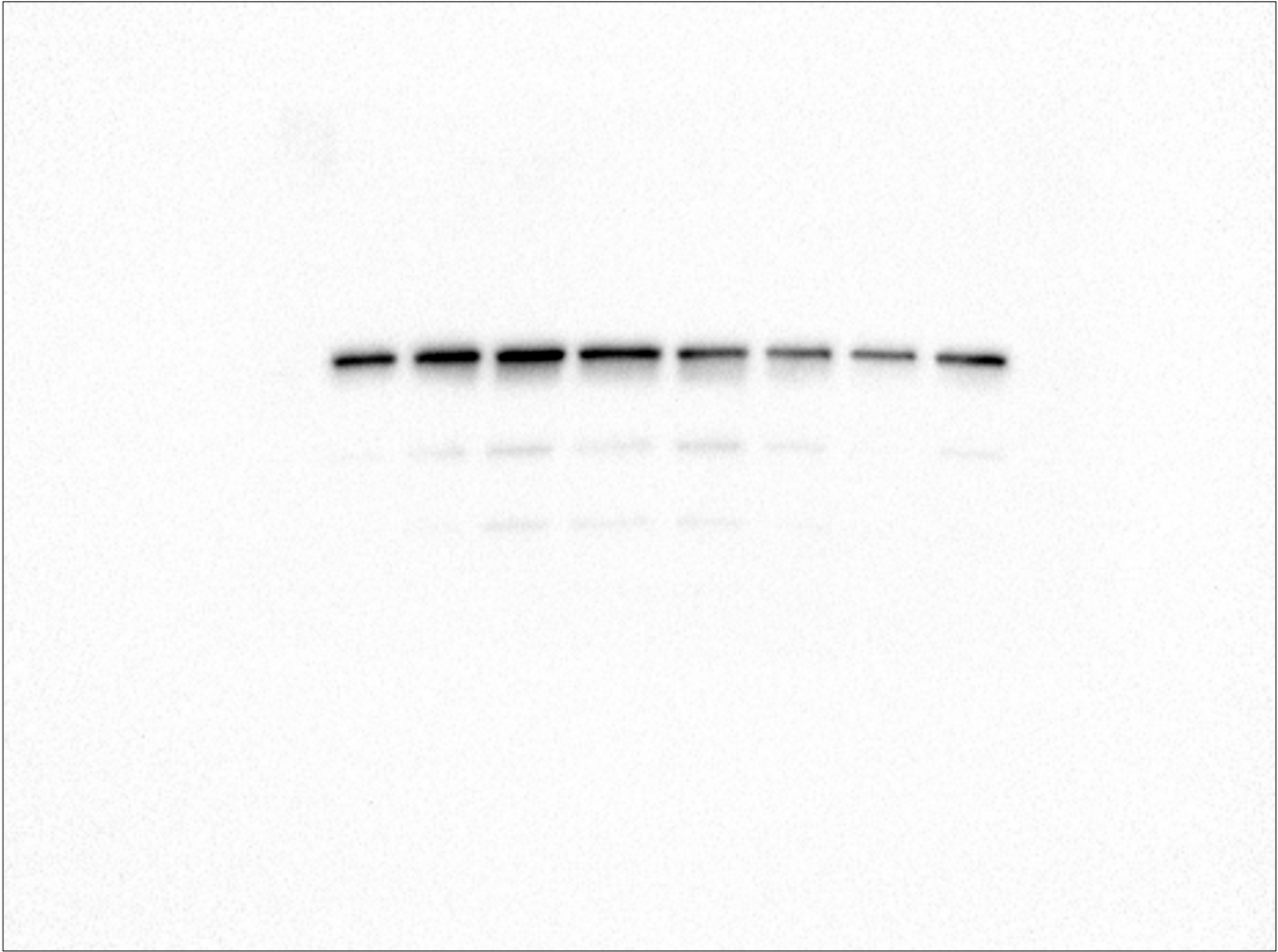

Supplement: Data S1. Raw images of Western Blot [file mmc7.zip › Western Blot/Fig5/Atub Left GSC Right DGC.pdf]

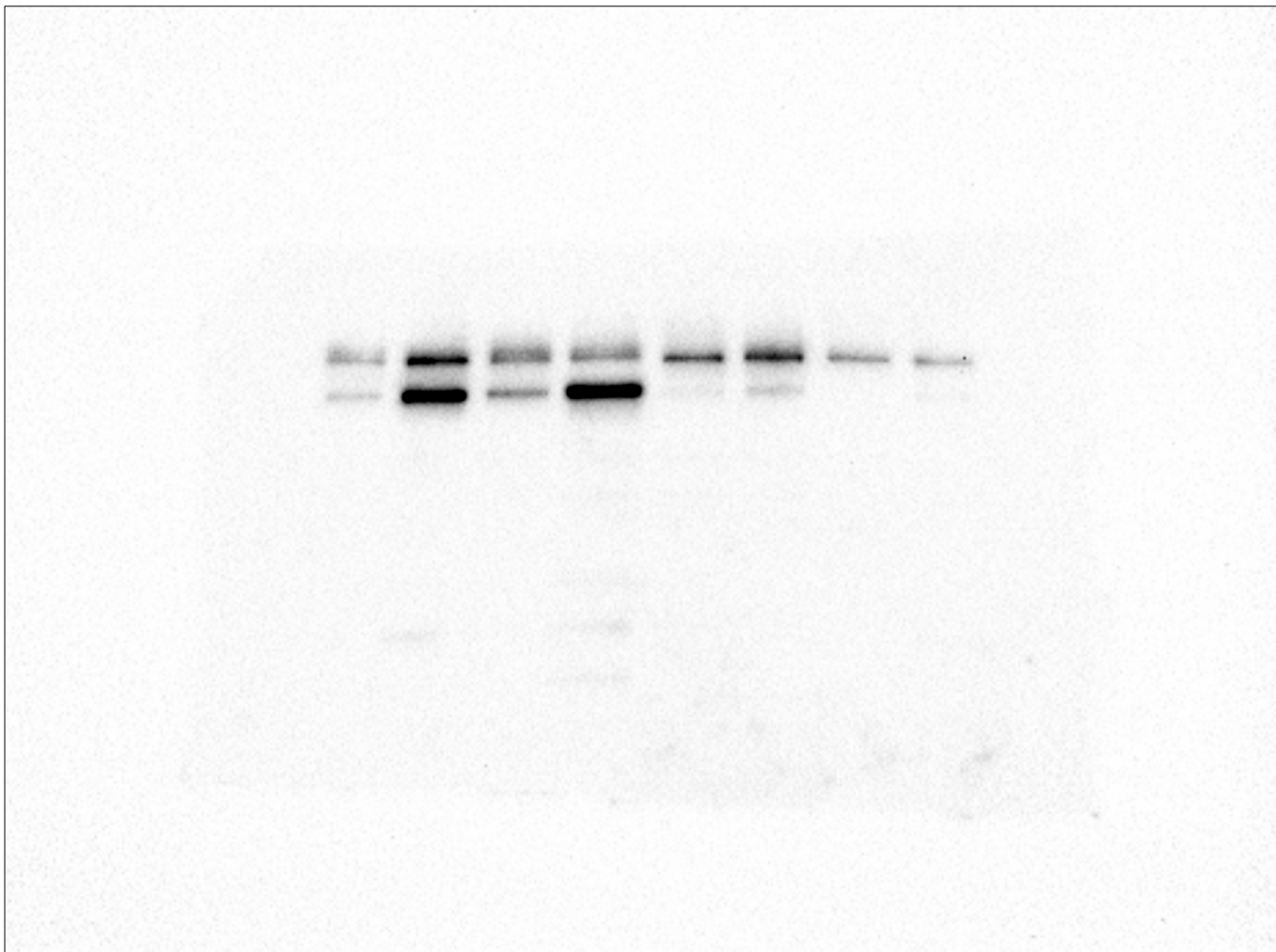

Supplement: Data S1. Raw images of Western Blot [file mmc7.zip › Western Blot/Fig5/PARP Left GSC Right DGC.pdf]

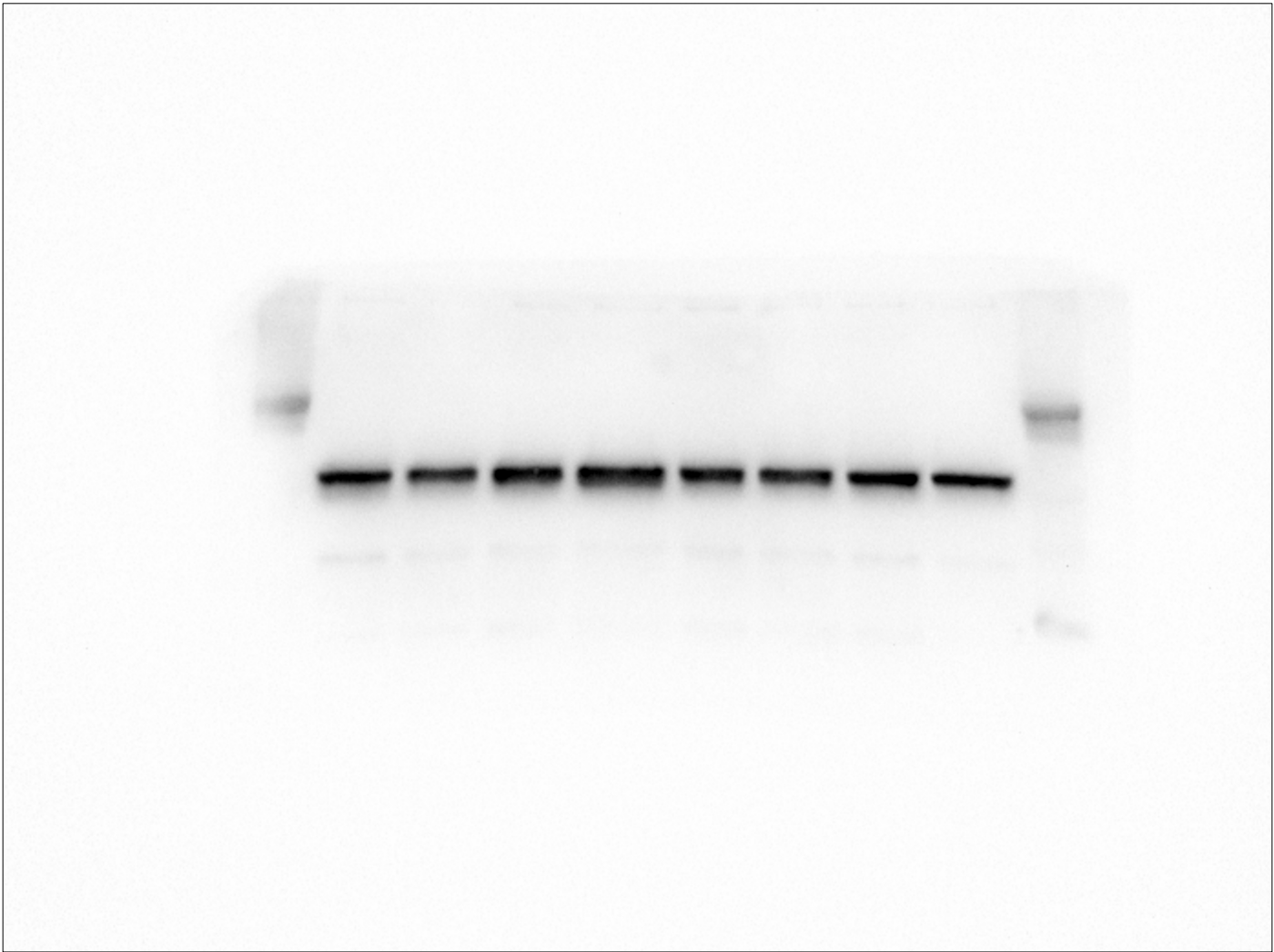

Supplement: Data S1. Raw images of Western Blot [file mmc7.zip › Western Blot/FigS4/CyclinB1 pCDK1 aTub/aTub MGG4 Left GSC Right DGC.pdf]

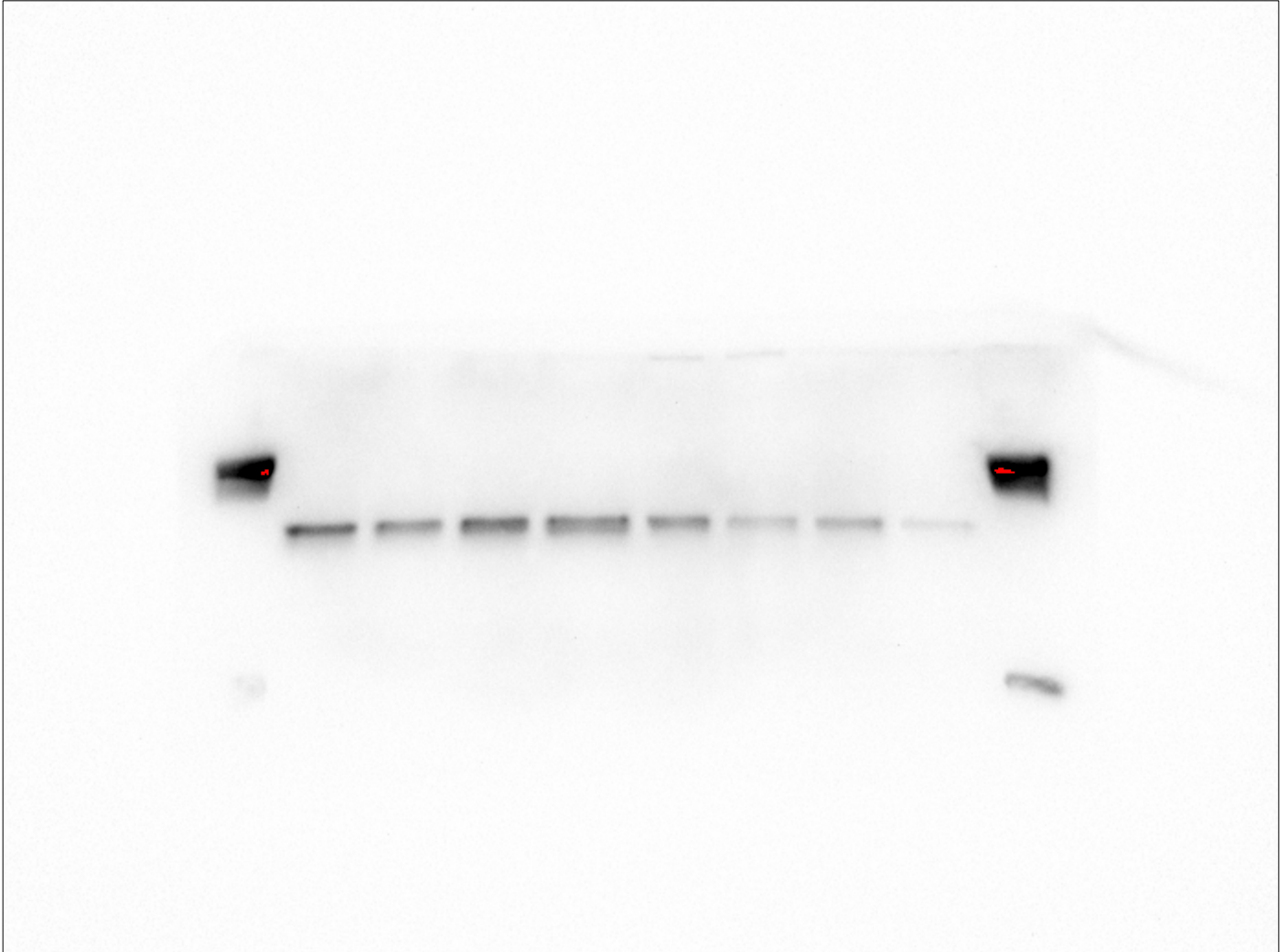

Supplement: Data S1. Raw images of Western Blot [file mmc7.zip › Western Blot/FigS4/CyclinB1 pCDK1 aTub/CiclinB1 MGG4 Left GSC Right DGC.pdf]

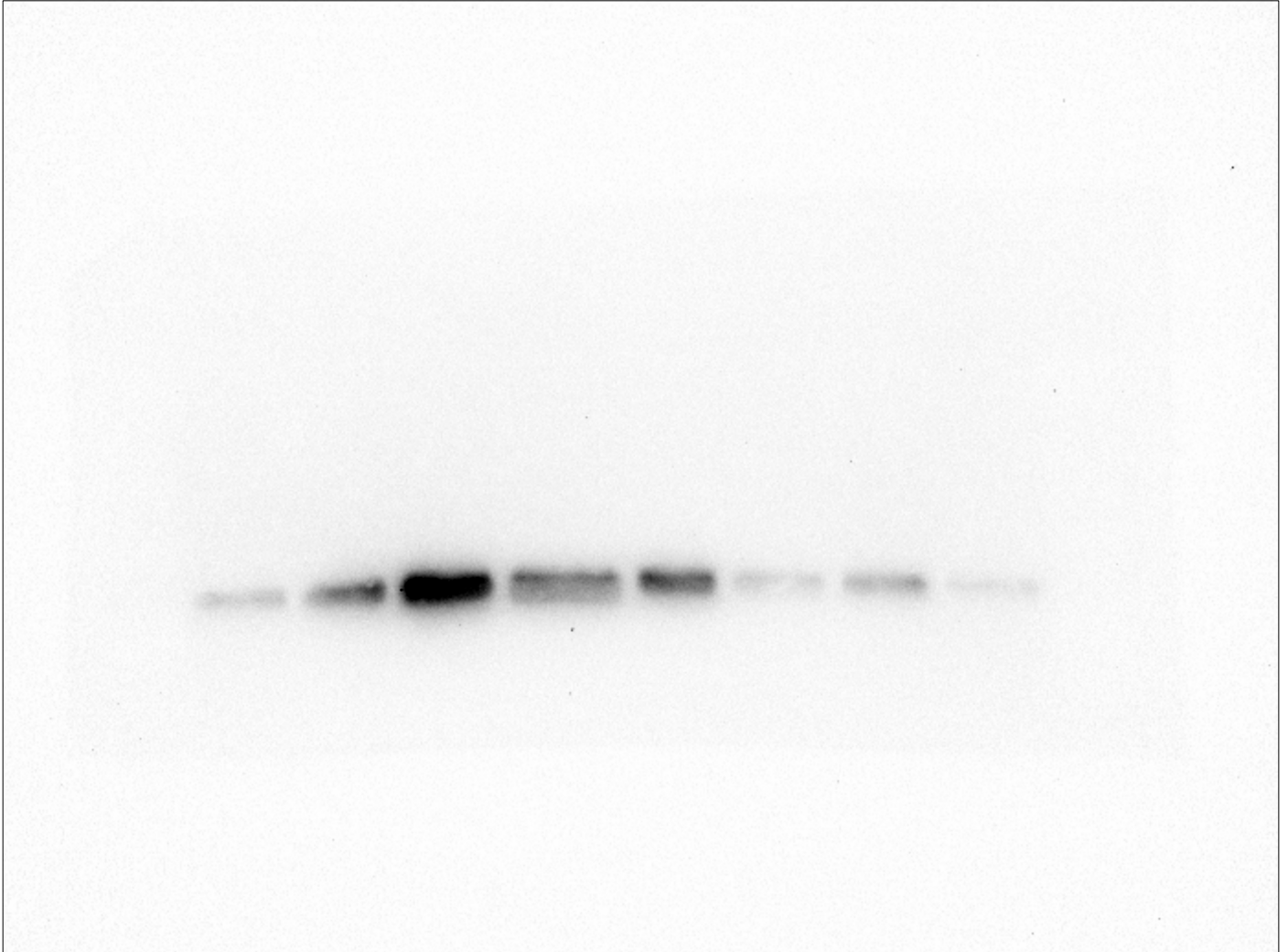

Supplement: Data S1. Raw images of Western Blot [file mmc7.zip › Western Blot/FigS4/CyclinB1 pCDK1 aTub/pCDK1 MGG4 Left GSC Right DGC.pdf]

2 Akt MGG4 20sec

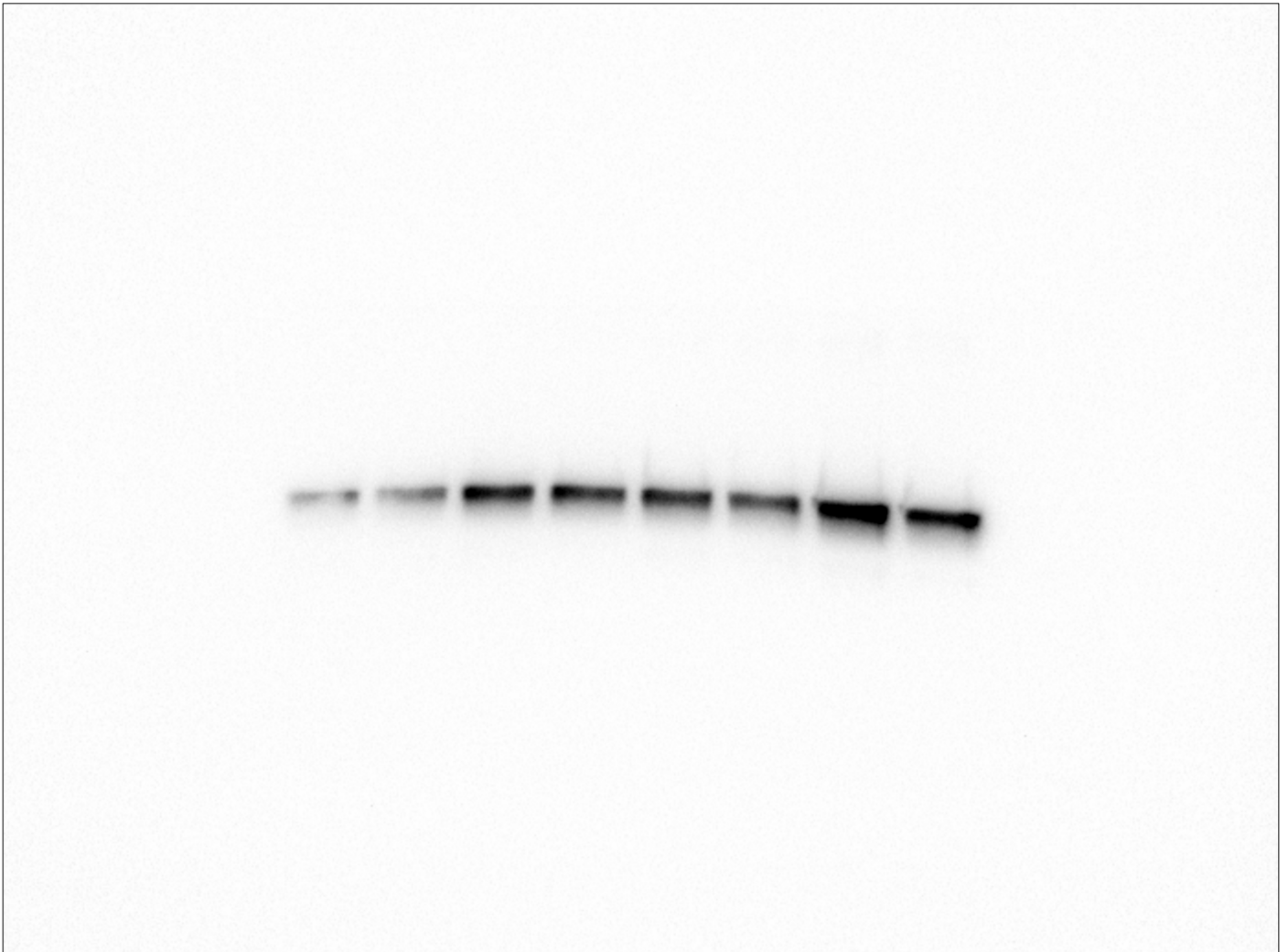

Supplement: Data S1. Raw images of Western Blot [file mmc7.zip › Western Blot/FigS4/pAkt Akt aTub/Akt MGG4 Left GSC Right DGC.pdf]

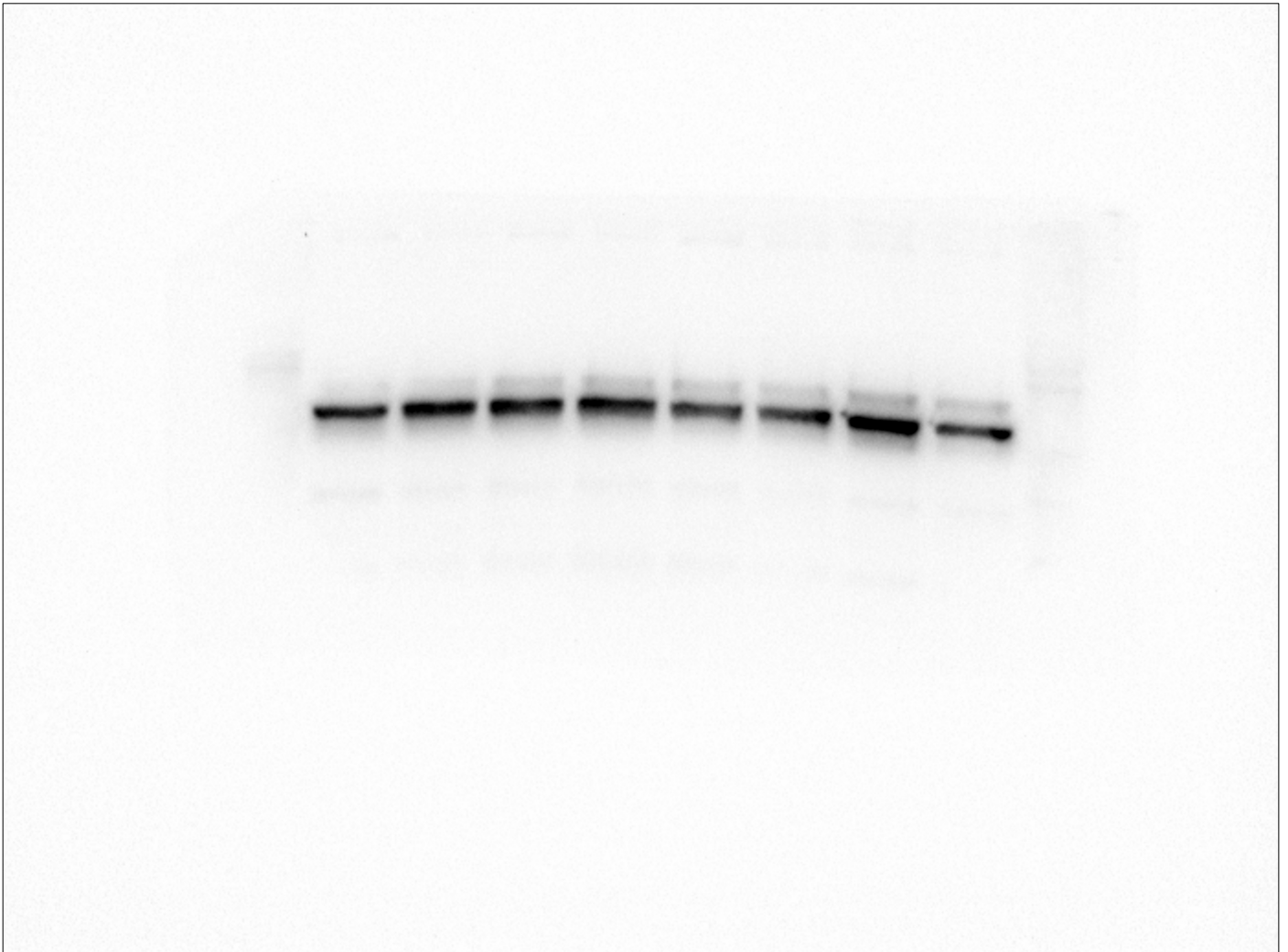

Supplement: Data S1. Raw images of Western Blot [file mmc7.zip › Western Blot/FigS4/pAkt Akt aTub/aTub MGG4 Left GSC Right DGCpdf.pdf]

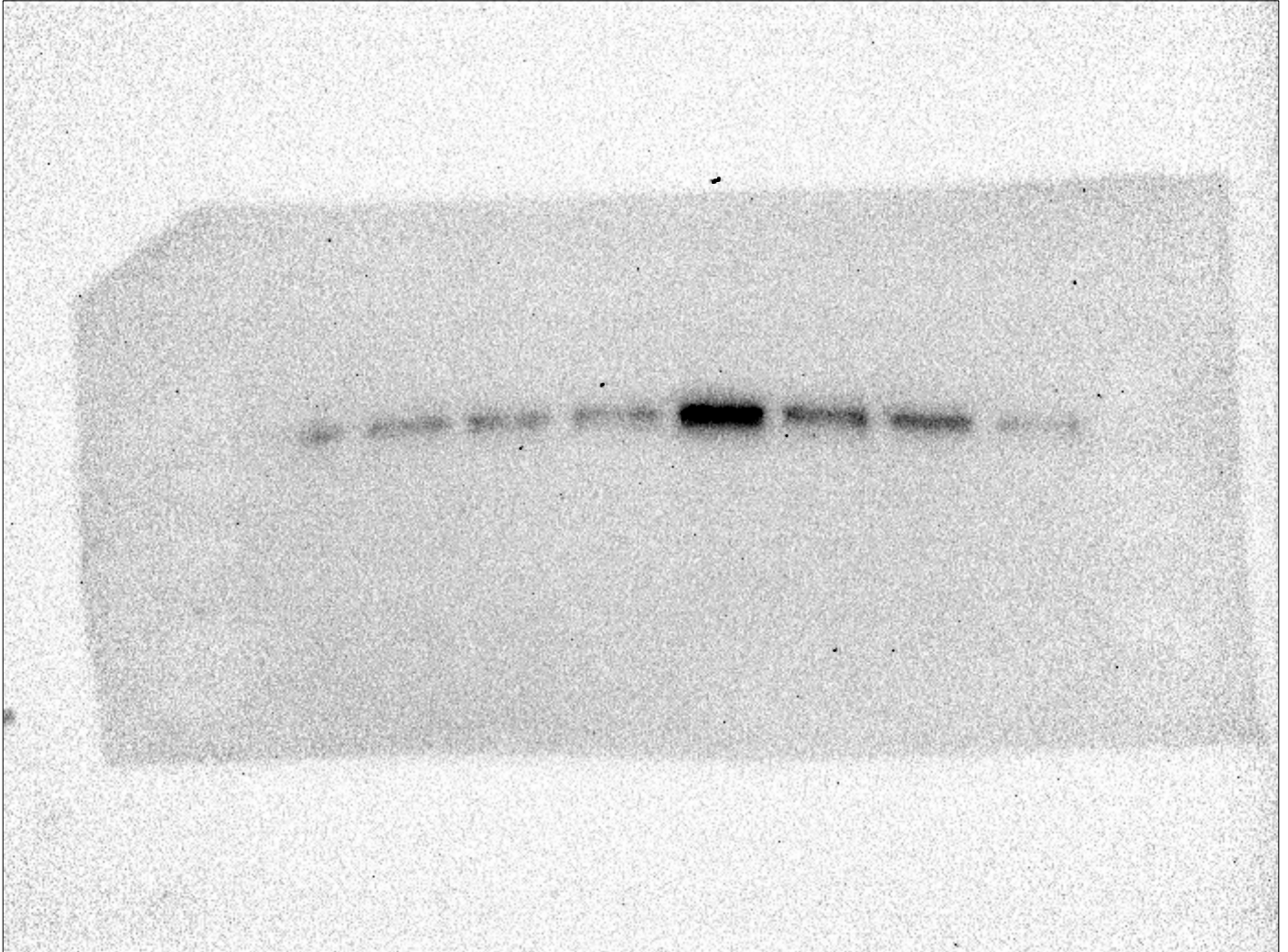

Supplement: Data S1. Raw images of Western Blot [file mmc7.zip › Western Blot/FigS4/pAkt Akt aTub/pAkt MGG4 Left GSC Right DGC.pdf]

αTubulin

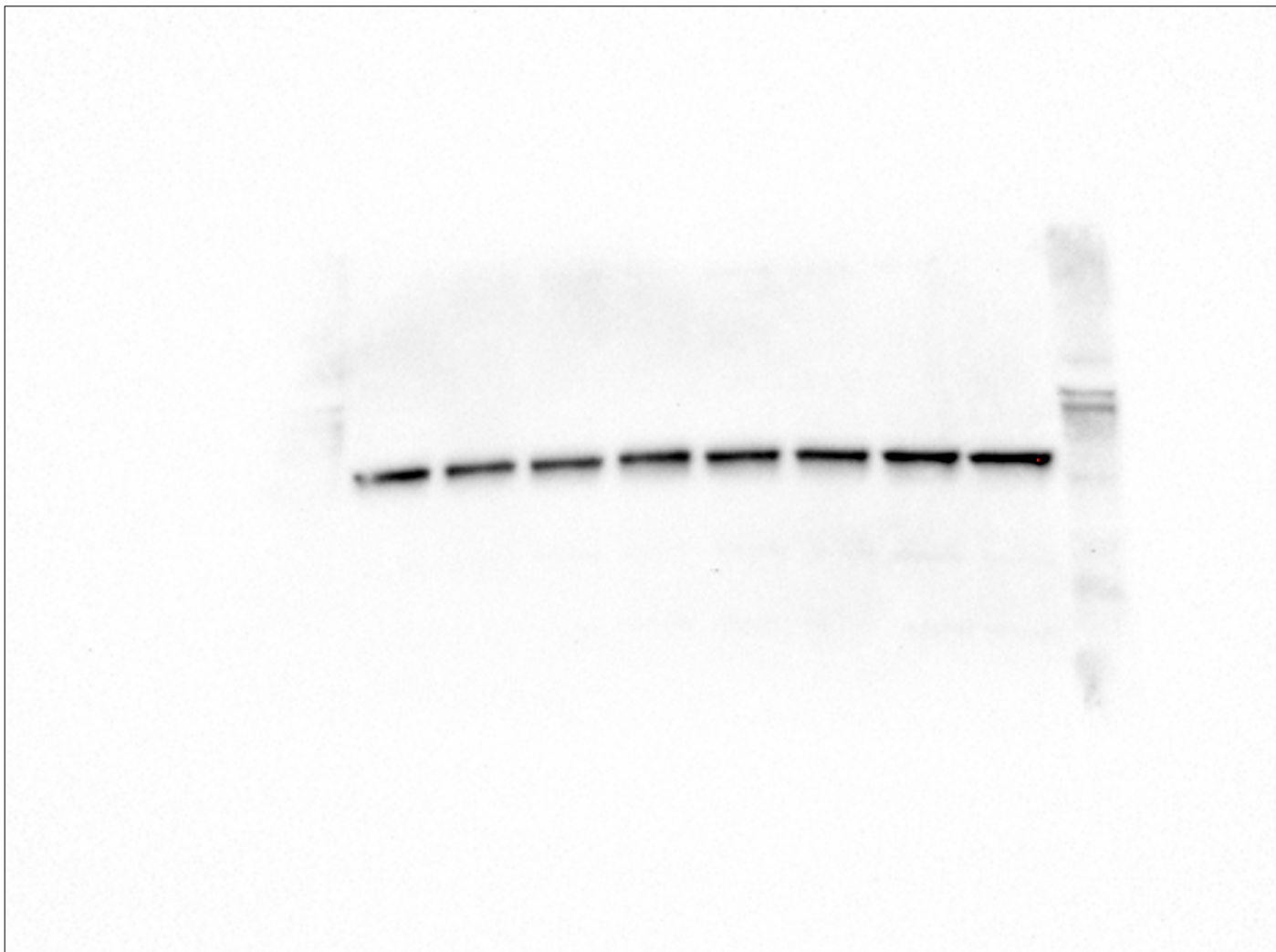

Supplement: Data S1. Raw images of Western Blot [file mmc7.zip › Western Blot/FigS5/cPARP PARP aTub/aTub MGG4 Left GSC Right DGC.pdf]

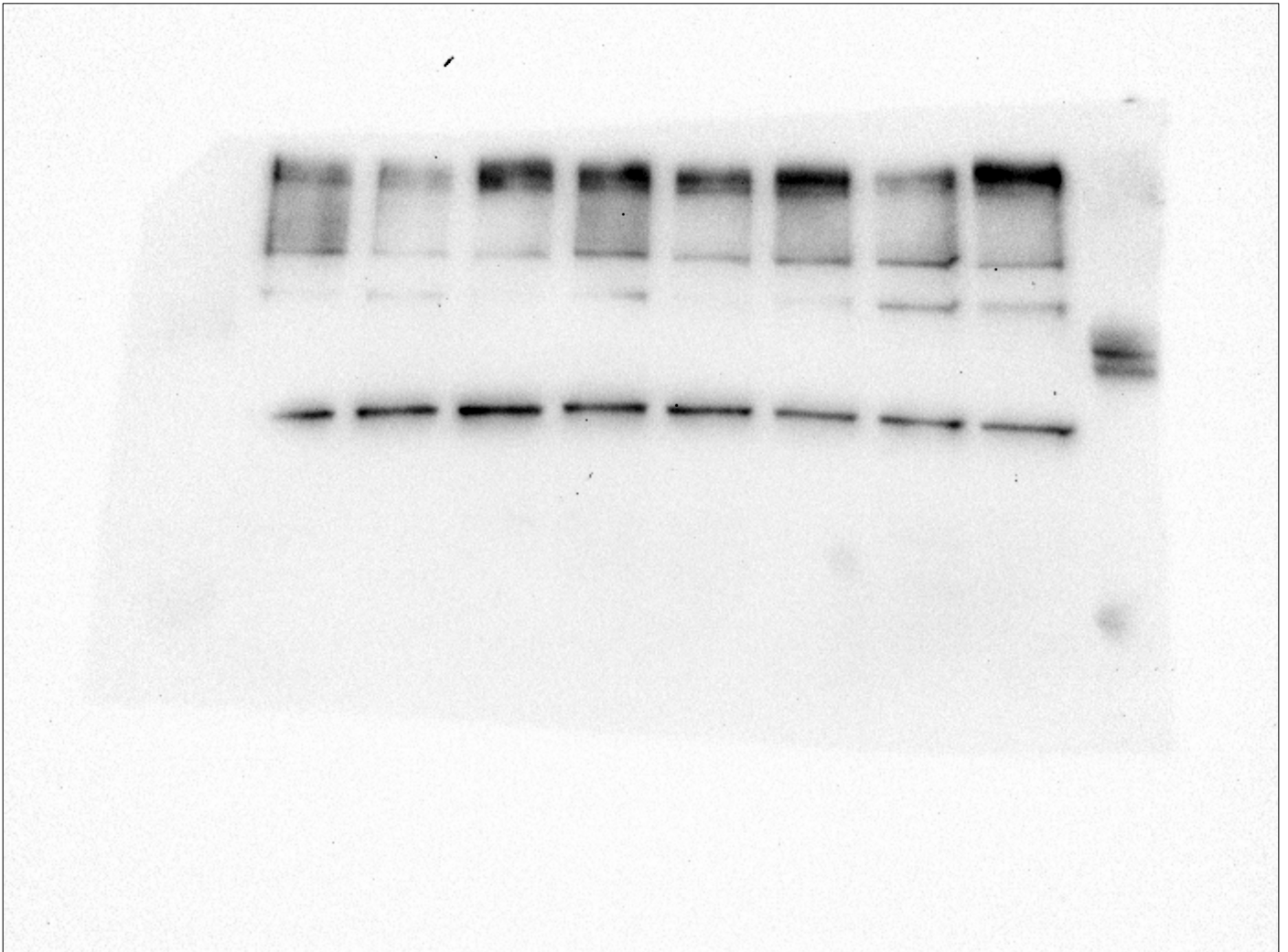

Supplement: Data S1. Raw images of Western Blot [file mmc7.zip › Western Blot/FigS5/cPARP PARP aTub/cPARP PARP MGG4 Left GSC Right DGC.pdf]
